# Cell Cycle, Filament Growth and Synchronized Cell Division in Multicellular Cable Bacteria

**DOI:** 10.3389/fmicb.2021.620807

**Published:** 2021-01-27

**Authors:** Nicole M. J. Geerlings, Jeanine S. Geelhoed, Diana Vasquez-Cardenas, Michiel V. M. Kienhuis, Silvia Hidalgo-Martinez, Henricus T. S. Boschker, Jack J. Middelburg, Filip J. R. Meysman, Lubos Polerecky

**Affiliations:** ^1^Department of Earth Sciences, Utrecht University, Utrecht, Netherlands; ^2^Department of Biology, University of Antwerp, Antwerp, Belgium; ^3^Department of Biotechnology, Delft University of Technology, Delft, Netherlands

**Keywords:** cable bacteria, stable isotope probing, nanoSIMS, filament growth, cell cycle, cell division

## Abstract

Cable bacteria are multicellular, Gram-negative filamentous bacteria that display a unique division of metabolic labor between cells. Cells in deeper sediment layers are oxidizing sulfide, while cells in the surface layers of the sediment are reducing oxygen. The electrical coupling of these two redox half reactions is ensured via long-distance electron transport through a network of conductive fibers that run in the shared cell envelope of the centimeter-long filament. Here we investigate how this unique electrogenic metabolism is linked to filament growth and cell division. Combining dual-label stable isotope probing (^13^C and ^15^N), nanoscale secondary ion mass spectrometry, fluorescence microscopy and genome analysis, we find that the cell cycle of cable bacteria cells is highly comparable to that of other, single-celled Gram-negative bacteria. However, the timing of cell growth and division appears to be tightly and uniquely controlled by long-distance electron transport, as cell division within an individual filament shows a remarkable synchronicity that extends over a millimeter length scale. To explain this, we propose the “oxygen pacemaker” model in which a filament only grows when performing long-distance transport, and the latter is only possible when a filament has access to oxygen so it can discharge electrons from its internal electrical network.

## Introduction

Cable bacteria are multicellular, filamentous bacteria that gain metabolic energy by coupling the oxidation of sulfide (H_2_S + 4 H_2_O → SO_4_^2–^ + 8 e^–^ + 10 H^+^) in deeper sediment layers to the reduction of oxygen (O_2_ + 4 H^+^ + 4 e^–^ → 2 H_2_O) at the sediment-water interface (Nielsen et al., 2010; Pfeffer et al., 2012). A remarkable aspect is that these two redox half-reactions occur in different cells of a given filament: “anodic” cells in deeper anoxic sediment layers only perform sulfide oxidation, while “cathodic” cells in the oxic zone only perform oxygen reduction. The necessary electrical coupling between these two redox half reactions is ensured by the transport of electrons over centimeter-scale distances through a regularly spaced network of highly conductive fibers that run along the whole filament (Meysman et al., 2019; Thiruvallur Eachambadi et al., 2020). This spatial separation of redox half-reactions allows cable bacteria to harvest sulfide over a wider range of sediment depths, which gives them a competitive advantage over other, single-celled sulfide-oxidizing bacteria (Meysman, 2018). Since their discovery, cable bacteria have been found at the oxic-anoxic interface in a wide range of aquatic sediment environments, including marine (e.g., Malkin et al., 2014; Burdorf et al., 2017), freshwater (Risgaard-Petersen et al., 2015), and aquifer (Müller et al., 2016) sediments. In these environments, cable bacteria strongly influence the elemental cycling of sulfur, iron, phosphorus, and methane (Risgaard-Petersen et al., 2012; Seitaj et al., 2015; Sulu-Gambari et al., 2016; Scholz et al., 2020). Additionally, cable bacteria have been found attached to the anode of a benthic microbial fuel cell placed in anaerobic conditions (Reimers et al., 2017) or in association with oxygenated zones around plant roots (Scholz et al., 2019) and worm tubes in marine sediments (Aller et al., 2019).

Cable bacteria belong to the family of *Desulfobulbaceae* (Trojan et al., 2016), which also contains single-celled sulfate-reducing and sulfur disproportionating bacteria. Genomic analysis suggests that cable bacteria oxidize sulfide by reversing the canonical sulfate reduction pathway and use the Wood–Ljungdahl pathway for inorganic carbon uptake (CO_2_ fixation), but also have the potential to additionally assimilate organic carbon (Kjeldsen et al., 2019). Stable isotope probing (SIP) experiments using ^13^C-labeled CO_2_ and propionate followed by either community lipid analysis (Vasquez-Cardenas et al., 2015) or analysis of individual cells and filaments by nanoscale secondary ion mass spectrometry (nanoSIMS) (Geerlings et al., 2020) have confirmed that cable bacteria incorporate both inorganic and organic carbon. Cable bacteria can thus be categorized as facultative chemoautotrophs (Vasquez-Cardenas et al., 2015; Kjeldsen et al., 2019; Geerlings et al., 2020). Interestingly, carbon fixation in cable bacteria appears to be strongly dependent on the redox environment, where only the sulfide-oxidizing cells assimilate carbon whereas the oxygen-reducing cells do not assimilate carbon (Geerlings et al., 2020). Thus, the dichotomy that characterizes the energy metabolism in cable bacteria is also directly reflected in their carbon metabolism. Consequently, it appears that the cathodic cells dispense electrons as quickly as possible via oxygen reduction without any energy conservation, while biosynthesis and growth remain restricted to the anodic cells, which are able to generate metabolic energy from sulfide oxidation (Kjeldsen et al., 2019; Geerlings et al., 2020).

A cable bacterium filament is linear (not branched) and typically consists of thousands of cells. Although the cells are separated from each other by a rigid septum, they share a periplasmic space that contains the network of conductive fibers, which run along the longitudinal axis of the filament (Pfeffer et al., 2012; Jiang et al., 2018; Meysman et al., 2019) and are inter-connected between adjacent cells by a cartwheel-shaped structure located within the septum (Cornelissen et al., 2018; Thiruvallur Eachambadi et al., 2020).

Cable bacterium filaments hence display a complex metabolism and architecture, but little is presently known about how these filaments grow and elongate. Previous observations by fluorescence microscopy have indicated that filament growth is too fast to be exclusively apical, and hence cell division must occur continuously along the filament (Schauer et al., 2014). Here, we combine SIP-nanoSIMS, fluorescence microscopy and genomic data to gain insights into the cell cycle of cable bacteria and the process of filament elongation. Previously, the SIP-nanoSIMS technique has shown that the rates of inorganic carbon and nitrogen assimilation are remarkably homogeneous among the cells of individual filaments that perform the sulfide-oxidizing half-reaction (Geerlings et al., 2020). Here, we use these previously published data and expand it with three-dimensional reconstructions of stable isotope incorporation to gain more detailed insights into the biomass synthesis and growth of cable bacteria. We show that, on the level of individual cells, the process of cell division in cable bacteria appears to be highly comparable to that of the Gram-negative model species *Escherichia coli.* Yet, on the filament level, cable bacteria display unique characteristics, where the cells performing sulfide oxidation show synchronized cell division along the filament over millimeter-scale lengths. We propose a model that links the observed synchronized cell division to the unique electrogenic metabolism of the cable bacteria.

## Materials and Methods

### Cable Bacteria Culturing

Enrichment cultures were prepared from natural sediment collected from a creek bed in Rattekaai Salt Marsh (Netherlands; 51.4391°N, 4.1697°E). At this site, earlier studies have documented the presence of cable bacteria *in situ* (Malkin et al., 2014). After collection in the field, the sediment was stored anoxically in the laboratory until further handling. At the start of the enrichment incubation, sediment was sieved (500 μm mesh size) to remove fauna and large debris, homogenized, and subsequently re-packed in polycarbonate cores (inner diameter 5.2 cm) as described before (Malkin et al., 2014). The sediment cores were submerged in artificial seawater (ASW, salinity of 32, the *in situ* value) and incubated in the dark for several weeks until an active cable bacteria population developed. The seawater was bubbled with air to maintain 100% air saturation, and the temperature (20°C) and salinity were kept constant throughout the incubation.

Two separate enrichment cultures were prepared. The first enrichment culture consisted of four replicate cores and was prepared with sediment collected in the summer of 2016 and incubated for 54 days between September and December 2017. The second enrichment culture involved four replicate cores and was prepared with sediment collected in March 2019 and incubated for 26 days between September and October 2019.

### Microsensor Depth Profiling

Microsensor depth profiling (O_2_, H_2_S, and pH) was performed to monitor the geochemical fingerprint within sediment cores, and thus the developmental state and metabolic activity of the cable bacteria population (Meysman et al., 2015). The microsensor depth profiles were also used to delineate the oxic and suboxic zones in the sediment at the time of core sectioning (see below). Microelectrodes (tip diameter; O_2_: 50 μm, H_2_S: 100 μm, pH: 200 μm) were purchased from Unisense A/S (Denmark), connected to a four-channel Microsensor Multimeter (Unisense), and mounted in a two-dimensional micro-profiling system that enabled stepwise movement of sensors. The SensorTrace PRO software (Unisense) was used to control the movement of the microsensors and record sensor signals. A general-purpose reference electrode (REF201 Red Rod electrode; Radiometer Analytical, Denmark) was used as reference during the pH measurements. Calibration of O_2_, H_2_S, and pH microsensors was performed as described in Malkin et al. (2014).

### Stable Isotope Probing

Independent stable isotope probing (SIP) experiments were conducted with each of the two enrichment cultures. For the first SIP experiment, two stock solutions were prepared, one containing 62 mM ^13^C-bicarbonate and 0.40 mM ^15^N-NH_4_, and the other containing 11 mM ^13^C-propionate (^13^C atom fraction 99%, all C-atoms labeled) and 0.40 mM ^15^N-NH_4_. For the second SIP experiment only one stock solution containing 62 mM ^13^C-bicarbonate and no added NH_4_ was used. Stock solution concentrations were chosen because they were successful in previous SIP experiments (Vasquez-Cardenas et al., 2015). Stock solutions used artificial seawater that contained no Mg^2+^ and Ca^2+^ to avoid precipitation of Mg^13^CO_3_ and Ca^13^CO_3_, and also no bicarbonate and ammonium ions to avoid label dilution. The salts (NaH^13^CO_3_, Na^13^CH_3_^13^CH_2_^13^COO, and ^15^NH_4_NO_3_) used for preparing the stock solutions were purchased from Sigma-Aldrich (Cas-numbers: 87081-58-1, 152571-51-2, and 31432-48-1, respectively).

Labeling of sediment cores was done by first inserting three subcores (inner diameter 1.2 cm) into a single enrichment culturing core with minimal disturbance of the sediment, and then injecting 500 μL of the labeled stock solution into each sub-core in ten separate 50 μL injections. To ensure homogeneous spread of the label throughout the sediment, the syringe needle was inserted from the top to a depth of 5 cm, and the 50 μL liquid was released while slowly retracting the needle upward. The subcores ensured that the label was spread within a well-constrained volume. At the end of the SIP incubation period (duration see below), the three subcores were extracted from the sediment core and sectioned. One sub-core was used to retrieve cable bacteria for SEM and NanoSIMS imaging, while the other two were used for porewater analyses.

To reduce loss of labeled CO_2_ to the atmosphere, SIP incubations were done by placing sediment cores in a sealed container filled with air. The bottom of the containers was covered with a thin layer of labeled ASW (label concentrations were the same as those of the porewater) to allow for gas exchange between this water and the overlying atmosphere, so the atmospheric CO_2_ in the container was also labeled. This procedure prevented water evaporation from the core and ensured similar ^13^C and ^15^N labeling of the overlying layer of water (∼2 mm) and porewater in the core (no label loss due to outgassing). The SIP incubation time in the first experiment was 24 h. This incubation time was chosen because previous experiments showed that the doubling time of cable bacteria is around 20 h (Schauer et al., 2014; Vasquez-Cardenas et al., 2015). The second SIP experiment was conducted for 6 h (two cores) and 24 h (two cores). Temperature was kept constant at 20°C in both SIP experiments.

### Filament Retrieval From the Sediment

At the end of the SIP incubation, cable bacterium filaments were isolated from the sediment matrix under a stereo microscope with fine glass hooks custom-made from Pasteur pipettes. Filaments were retrieved separately from the oxic (0–2 mm depth) and the middle of the suboxic (5–10 mm depth) zone of the sediment. Isolated filaments were washed several times (>3) in Milli-Q water (Millipore, Netherlands) to eliminate precipitation of salt, transferred onto polycarbonate filters (pore size 0.2 μm; Isopore, Millipore, Netherlands) pre-coated with a ∼5 nm thin gold layer, and air-dried in a desiccator for ∼24 h.

### Scanning Electron Microscopy (SEM)

Filaments on the polycarbonate filters were imaged with a scanning electron microscope (JEOL Neoscope II JCM-6000, Japan) to identify filament sections suitable for NanoSIMS analysis. Imaging was done under a 0.1–0.3 mbar vacuum and a high accelerating voltage (15 kV) using a backscattered electron detector.

### NanoSIMS Analysis

Analysis by nano-scale secondary ion mass spectrometry (nanoSIMS) was performed with the nanoSIMS 50L instrument (Cameca, France) operated at Utrecht University. Fields of view (FOV) selected through SEM were pre-sputtered with Cs^+^-ions until secondary ion yields stabilized. Subsequently the primary Cs^+^-ion beam (current: 0.5–2 pA, energy: 16 keV, beam size: 130 nm) was scanned over the FOV (areas between 10 × 10 μm and 20 × 20 μm in size, dwell time: 1–2 ms per pixel) while detecting secondary ions ^12^C^–^, ^13^C^–^, ^12^C^14^N^–^, ^12^C^15^N^–^, ^31^P^–^, and ^32^S^–^. In some samples the ^12^C^14^N^–^/^13^C^14^N^–^ ion pair was measured instead of the ^12^C^–^/^13^C^–^ ion pair.

NanoSIMS analysis of most samples focused on the variation of the mean isotopic and elemental composition among cells within filaments. In these analyses the same FOV was imaged multiple times (180–300 frames) and the resulting ion count images were aligned and accumulated. For some samples we aimed to obtain additional insight into the 3D distribution of the isotopic and elemental composition within cells. These measurements were therefore conducted over a substantially larger number of frames (up to 7000) until the sample material was completely sputtered away by the primary ion beam.

NanoSIMS data were processed using the Matlab-based software Look@NanoSIMS (Polerecky et al., 2012). After alignment and accumulation of the measured planes, regions of interest (ROIs), which corresponded to single cells, were drawn manually using the combined ^12^C^14^N^–^ and ^31^P^–^ ion count images. For each cell, the cell-specific ^13^C atom fraction was calculated using the total counts of the ^12^C^–^ and ^13^C^–^ ions (or ^12^C^14^N^–^ and ^13^C^14^N^–^ ions) accumulated over all ROI pixels. Similarly, the ROI-specific ^15^N atom fraction was calculated from the total counts of the ^12^C^14^N^–^ and ^12^C^15^N^–^ ions accumulated over all ROI pixels. ROIs were excluded from the analyses if the cells appeared damaged.

To gain insight into the 3D distribution of the isotopic and elemental composition within cells, the Look@NanoSIMS program was updated by adding a new feature that allows visualization of the depth variation in the nanoSIMS data (ion counts or ion count ratios) along a lateral profile. A more detailed description of this feature is provided in the Supplementary Methods.

In the first SIP experiment (^13^C and ^15^N labeling for 24 h) a total of 596 cells from 21 filaments were analyzed by nanoSIMS. Distinct sections (10–15 cells) were analyzed in each filament, separated by distances that ranged between 169–4,845 μm (Table 1 and Supplementary Figure 1). Eleven filaments originated from the incubation with ^13^C-bicarbonate (3 and 8 filaments from the oxic and suboxic zone, respectively), and 10 filaments from the incubation with ^13^C-propionate (all from the suboxic zone).

**TABLE 1:** Cable bacteria filaments examined with nanoSIMS at multiple areas along the length: measured length, maximum length between measurements, number of measured cells and the mean ^13^C and ^15^N atom ratios and their corresponding standard deviations, the median values, mean absolute deviation (MAD) and coefficient of variation (CV). Differently shaded rows correspond to different zones and cores from which the analyzed filaments were retrieved (see table footnote 1).

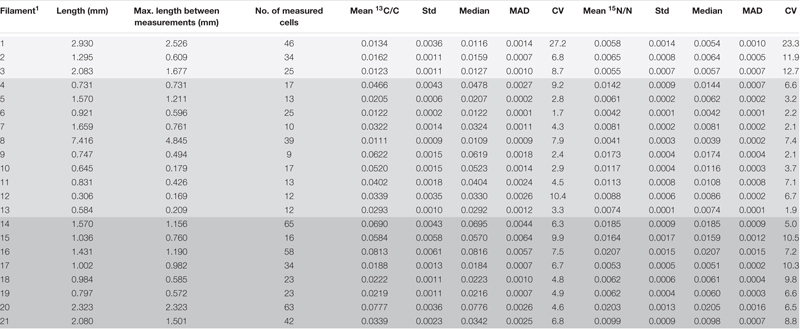

*^1^Filaments 1–3 were retrieved from the oxic zone of a core incubated for 24 h with ^13^C-bicarbonate and ^15^N-ammonia. Filaments 14–21 were retrieved from the suboxic zone of the same core. The ^13^C atom fraction of the DIC in the pore water after the incubation was 0.123. Filaments 4–13 were retrieved from a core incubated for 24 h with ^13^C-labeled propionate and ^15^N-ammonia. Due to remineralization of the propionate during the incubation, increasing levels of labeled ^13^C-bicarbonate were also available to the cable bacteria in this incubation. The ^13^C atom fraction of the DIC in the pore water at the end of the incubation was 0.0821.*

The within-cell heterogeneity was assessed in more detail and in 3D in 12 cells from 3 filaments; 8 cells from 2 filaments originating from the second SIP experiment (6 h incubation with ^13^C-labeled bicarbonate and without ^15^N-labeled ammonia) and 4 cells from one filament from the first SIP experiment (24 h incubation with ^13^C-labeled bicarbonate and ^15^N-labeled ammonia).

As previously shown (Geerlings et al., 2020), the majority of the ^13^C labeling in cells from the ^13^C-propionate incubation is due to the assimilation of inorganic ^13^C produced through mineralization of the added ^13^C-propionate, whereas propionate assimilation plays only a minor role in cable bacteria. In this study we refer to C assimilation without distinguishing between inorganic and organic C.

To assess the relative variability of ^13^C and ^15^N assimilation among cells within a filament and the variation of ^13^C and ^15^N among filaments the coefficient of variation (CV) was calculated for each of the individual filaments. To test whether the average ^13^C atom fraction measured in all cells within a filament varied significantly from the natural ^13^C atom fraction (0.011), a one-sample Wilcoxon test was performed because there were only 30 cells measured and the distribution of the data was non-normal.

### Pore Water Analysis

The ^13^C-labeling of the porewater dissolved inorganic carbon pool (DIC) was measured as described in Geerlings et al. (2020). Because of the limited porewater volume in the sampled sub-cores, these analyses could not be performed separately for the oxic and anoxic zones. When possible, the handling was done under CO_2_-free conditions (N_2_ atmosphere) to minimize exchange with atmospheric CO_2_. Under CO_2_-free conditions, the top 3 cm of the sub-cores were sliced off and transferred into a 50 mL Greiner tube. The sediment was then centrifuged at 3,000 rpm for 10 min. Subsequently, while still under anoxic conditions, the supernatant was retrieved and filtered over 0.45 μm pore size filters. Following filtration, 0.3 mL, 0.5 mL, or 0.7 mL of the filtered porewater were injected into helium-flushed (5 min, flush rate of 70 mL min^–1^) air-tight septum-capped vials (12 mL) that contained four drops of 85% H_3_PO_4_, which were subsequently analyzed by GasBench IRMS.

### Fluorescence Microscopy

A glass slide sandwich system was inserted into the sediment to observe cable bacterium filaments across the oxic-anoxic interface. To this end, two microscopy slides were pressed against each other and ASW was added in between. These “double slides” were then inserted half-way into the sediment of enrichment cultures (sieved 350 μm mesh) such that the longer edge was parallel to the sediment surface. This arrangement allowed the development of opposing gradients of sulfide and oxygen within the layer of ASW between the slides. After several weeks, when numerous cable bacteria filaments were observed between the slides, the slides were carefully separated, the bacteria were stained with the general DNA stain 4′,6-diamidino-2-phenylindole (DAPI), and a coverslip was placed on top and sealed with a nail polish. The stained filaments were then imaged using a Zeiss Axiovert 200M epifluorescence microscope (Carl Zeiss, Göttingen, Germany) equipped with the Zeiss filter set 02 (excitation G365, beamsplitter: BS395; emission LP420) and filter set 09 (excitation: BP450-490, beamsplitter: FT 510, emission: LP 515).

### Genome Analysis

The (draft) genome sequences of marine cable bacteria species *Candidatus* Electrothrix aarhusiensis and *Ca*. Electrothrix marina sp. A5, and of sulfate reducing *Desulfobulbus propionicus* and *Desulfobulbus japonicus* (all belonging to the family *Desulfobulbaceae* in the class Deltaproteobacteria), were examined for the presence of genes involved in cell division. The sulfate reducing representatives were selected because of their close phylogenetic proximity to the cable bacteria species. The genes examined include the genes that constitute the division and cell wall (*dcw*) cluster, as well as other genes known to be involved in cell division in the Gram-negative model organism *Escherichia coli* (class Gammaproteobacteria). The genomes and genome annotations were downloaded from GenBank^1^. For the two cable bacteria genomes, automatic genome annotation was also performed in RAST (Overbeek et al., 2014), using the classic RAST annotation scheme. Genes were identified by automatic annotation and blastp analysis. The predicted gene functions of all genes discussed were manually curated and revised as necessary by comparison to the NCBI, Pfam, and COG databases.

## Results

### C and N Assimilation at the Filament Level

**FIGURE 1:**
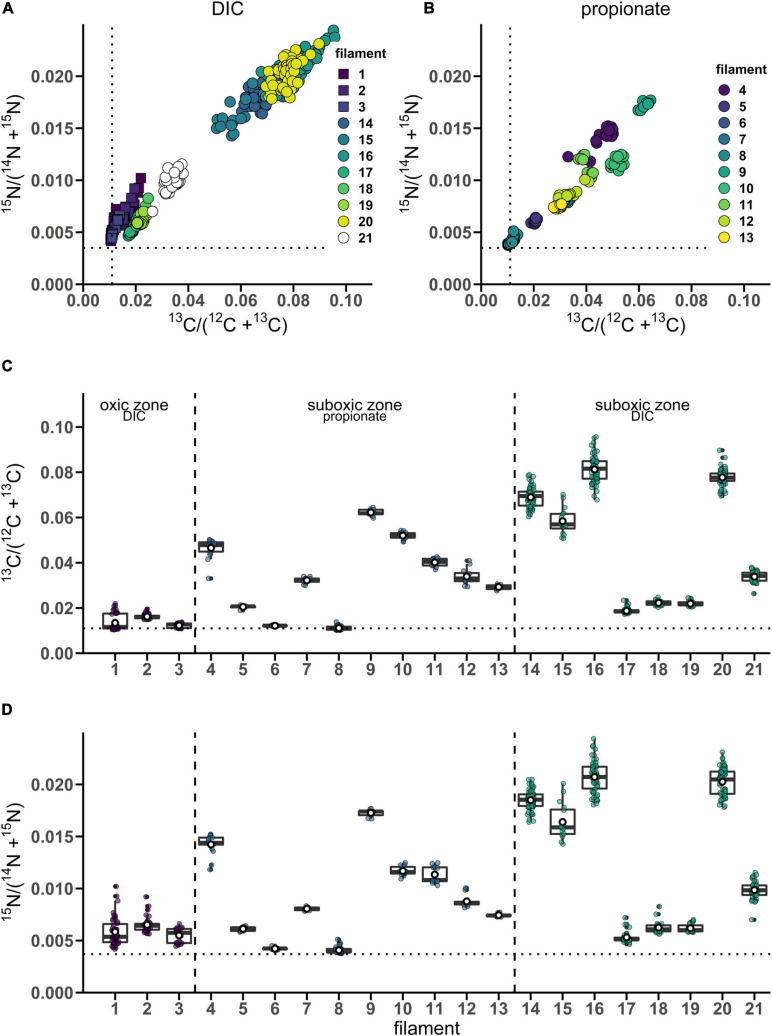
Variation of isotope label uptake among cells in individual cable bacteria as measured by nanoSIMS. Cross-plots show the correlation between the average ^13^C and ^15^N atom fractions per cell. Shown are atom fractions from 24 h-labeling with **(A)** ^13^C-bicarbonate and ^15^N-ammonia and **(B)** ^13^C-propionate and ^15^N-ammonia. Each data point represents the mean ^13^C and ^15^N atom fraction for a cell measured in the followed filaments. Colors and symbols differentiate between individual filaments and redox zones in the sediment from which the filaments were retrieved, respectively. Boxplots show the average **(C)** ^13^C or **(D)** ^15^N atom fraction in individual cells (data points) and the corresponding mean (white open dot), median (black line) and upper and lower quartiles for each individual filament. *n* = 21 filaments have been analyzed in total. Individual filaments were retrieved from the oxic zone of the incubation with ^13^C-labeled bicarbonate (*n* = 3), from the suboxic zone of the incubation with ^13^C-labeled propionate (*n* = 10) and from the suboxic zone of the incubation with ^13^C-labeled bicarbonate (*n* = 8). Dotted lines represent the natural ^13^C (0.011) and ^15^N (0.0037) atom fractions. The clustering of data points shows that intra-filament variation is substantially smaller than inter-filament variation. The filaments from the suboxic zone incubation with ^13^C-labeled bicarbonate (14–21) were also analyzed by Geerlings et al. (2020). The corresponding nanoSIMS images of the ^13^C atom fractions from each filament are given in Supplementary Figure 1 (filaments #1–19 and #21) or in Figure 5 (filament #20). The values of the ^13^C and ^15^N atom fractions for each of the cells can be found in Supplementary Datasheet 1.

From the 24 h labeling period with ^13^C-bicarbonate or ^13^C-propionate and ^15^N-ammonia, nanoSIMS data were obtained from 21 individual filaments (filament length ranged between 169 and 4,845 μm). The resulting data are similar between replicate cores and confirm three observations that were previously reported in Geerlings et al. (2020). Firstly, there was a strong linear correlation between ^13^C assimilation and ^15^N assimilation (Figures 1A,B). Secondly, when filaments showed a high label uptake, they were always retrieved from the suboxic zone (Figures 1C,D). Filaments retrieved from the oxic zone showed ^13^C and ^15^N atom fractions close to the natural abundance measured in control cells (0.011 and 0.0037, respectively). Thirdly, the data revealed a limited variability in the assimilation of ^13^C and ^15^N among cells within individual filaments, but strong differences in labeling among filaments (Figures 1A,B and Table 1). For example, the longest filament analyzed (#8; measured length of 4.845 μm) showed no significant ^13^C and ^15^N assimilation over the entire length (one sample Wilcoxon test; V = 443, *p* = 0.232), while another long filament (#20, measured length of 2.323 μm) showed high assimilation of both ^13^C and ^15^N (mean ^13^C atom fraction = 0.0777; mean ^15^N atom fraction = 0.0203) and a homogeneous label uptake among cells as indicated by small coefficients of variation (CV = 4.6% for the ^13^C atom fraction; CV = 6.5% for the ^15^N atom fraction; Table 1). In general the filaments showed little cell-to-cell variation in the label uptake (CV values ranging between 2 and 28% for the ^13^C atom fractions and 2–23% for the ^15^N atom fractions; Table 1).

### C Assimilation at the Single Cell Level

**FIGURE 2:**
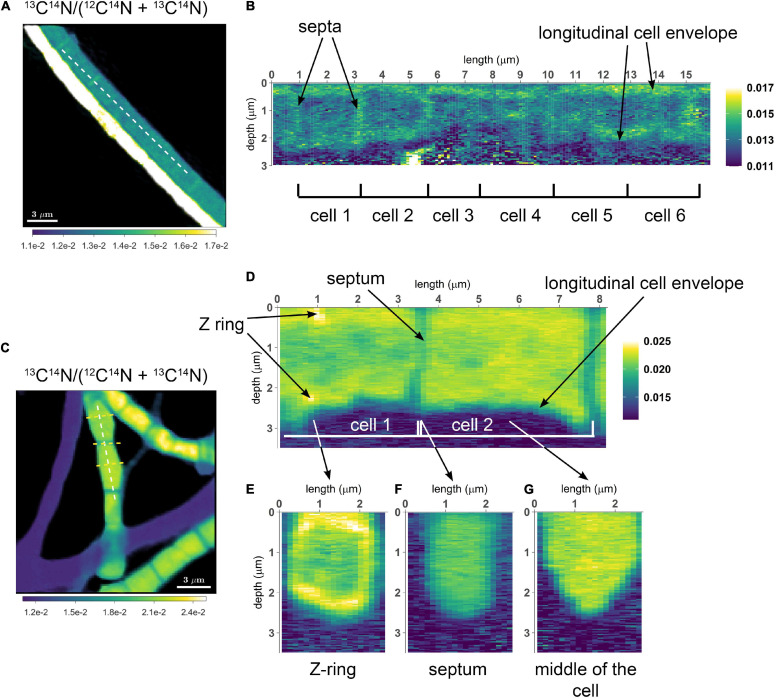
NanoSIMS images of cable bacterium filaments retrieved from the suboxic zone of the sediment. All images show the ^13^C atom fraction, calculated from the secondary ion count as ^13^C^14^N/(^12^C^14^N + ^13^C^14^N). Measurements were performed on filaments retrieved from an incubation that was labeled with ^13^C-bicarbonate for 6 h. **(A)** NanoSIMS image of two parallel filaments. The bottom filament is more strongly ^13^C labeled compared to the top filament. **(B)** A longitudinal cross section was analyzed over the length of six consecutive cells in the weakly labeled top filament [dotted line in panel **(A)**]. The image shows the ^13^C atom fraction as a function of depth. In this filament, increased ^13^C enrichment is observed in the cell envelope and cell junctions, whereas the cytoplasm shows a ^13^C atom fraction similar to the natural level (0.011). **(C)** NanoSIMS image of the ^13^C atom fraction showing three filaments with stronger ^13^C labeling (in addition to three filaments with no ^13^C enrichment). **(D)** A longitudinal depth analysis [white dotted line in panel **(C)**] shows the ^13^C atom fraction with depth along the length of two adjacent cells. The cell envelope and junction show less ^13^C enrichment than the cytoplasm. In cell 1, a ring with strong ^13^C enrichment is observed (interpreted here as a Z-ring). A depth analysis across three transverse cross-sections was also performed [yellow dotted lines in panel **(C)**]. **(E)** Transverse cross-section through the middle of a cell with a Z-ring. **(F)** Transverse cross-section through a cell-junction. **(G)** Transverse cross-section through the middle of a cell without a Z-ring. Here, the cytoplasm is more enriched in ^13^C than the surrounding cell envelope. The color scale in panel **(A,B)** was adjusted to highlight the isotope enrichment in the cell envelope. The color scale in panel **(E–G)** is the same as in panel **(C,D)**.

**FIGURE 3:**
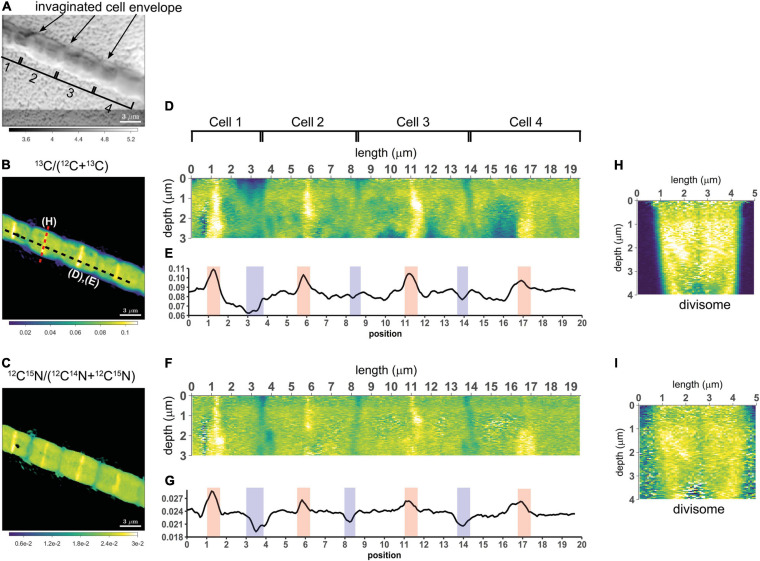
NanoSIMS images of a highly labeled filament in the process of cell division. Measurements were performed on a filament (#20) retrieved from the suboxic zone after labeling with ^13^C-bicarbonate and ^15^N-ammonium for 24 h. **(A)** Secondary electron image showing the four cells investigated. The invagination of the cell envelope indicates the cell septa (highlighted by the black arrows). **(B)** Corresponding nanoSIMS image of the ^13^C atom fraction, calculated as ^13^C/(^12^C + ^13^C). **(C)** Corresponding nanoSIMS image of the ^15^N atom fraction, calculated as ^12^C^15^N/(^12^C^14^N + ^12^C^15^N). The black dotted line in panel **(B)** shows where the longitudinal cross-section **(D,F)** and line analysis **(E,G)** were performed (19.5 μm in total). **(D,F)** The longitudinal cross-sections show the ^13^C atom fraction **(D)** and the ^15^N atom fraction **(F)** as a function of depth. **(E,G)** The line analysis shows the depth-averaged ratios of the ^13^C atom fraction **(E)** and ^15^N atom fraction **(G)** along the length of four cells. The red and blue areas show the middle of the dividing cell and the cell septa, respectively. **(H,I)** Transverse cross-section depicting the ^13^C atom fraction **(H)** and the ^15^N atom fraction **(I)** as a function of depth. The cross-section was taken at a division plane [red dotted line in panel **(B)**]. Color scales for panel **(D,H)** are the same as for panel **(B)**. Color scales for panel **(F,I)** are the same as for panel **(C)**.

To assess the variation of C assimilation within a cell, fragments comprising a few cells of three different filaments from the 6 h labeling period (only ^13^C-bicarbonate) and one filament from the 24 h labeling period (^13^C-bicarbonate and ^15^N-ammonia, filament #20) were investigated in more detail. At the single cell level, cells showed marked differences in the degree of ^13^C labeling of the cytoplasm versus the cell envelope (here defined as encompassing both the cell septa and the longitudinal cell wall). In effect, cells revealed three distinct intracellular isotopic labeling patterns, which were dependent on the degree to which a given cell was labeled in ^13^C. (1) At lowest levels of ^13^C labeling (^13^C/C only slightly above the natural abundance), the cell envelope was typically more enriched in ^13^C than the cytoplasm (Figures 2A,B). (2) At intermediate ^13^C enrichments (^13^C/C ∼ 0.02–0.06), the cytoplasm became more enriched in ^13^C than the cell envelope (Figures 2C–G). In one occasion, a locally pronounced ^13^C enrichment was observed in the middle section of the cell (Figure 2D), and a transversal cross-section demonstrated that this elevated ^13^C formed a ring at the periphery of the cytoplasm (Figure 2E). (3) At highest levels of ^13^C enrichment (^13^C/C ∼ 0.06–0.08), the cytoplasm is again more enriched in ^13^C than the cell envelope (Figure 3, filament #20). Likewise, a locally more pronounced ^13^C enrichment is observed in a thin band (∼300 nm) through the middle of the cell (Figures 3A,B). However, transversal cross-section analysis revealed that this region now extends through the entire cross-section of the cell, and thus forms a disk rather than a ring (Figures 3D,H). When the isotope label incorporation is averaged in the vertical direction and plotted along the longitudinal axis of the filament, the ^13^C enrichment shows a regular alternating pattern: lower levels at the cell septa and higher levels in the middle of the cell relative to the level in the cytoplasm (Figure 3E). The depth-averaged ^13^C atom fraction observed in the middle of these four cells (^13^C/C = 0.100 ± 0.002) was close to the ^13^C atom fraction of the pore water DIC pool (^13^C/C = 0.112), thus indicating that most of the carbon in these disks was newly assimilated during the labeling period. In all cases, the spatial patterns in ^15^N labeling were highly similar to those observed in ^13^C (Figures 3C,F,G,I).

### Synchronized Cell Division Along a Filament in the Suboxic Zone

**FIGURE 4:**
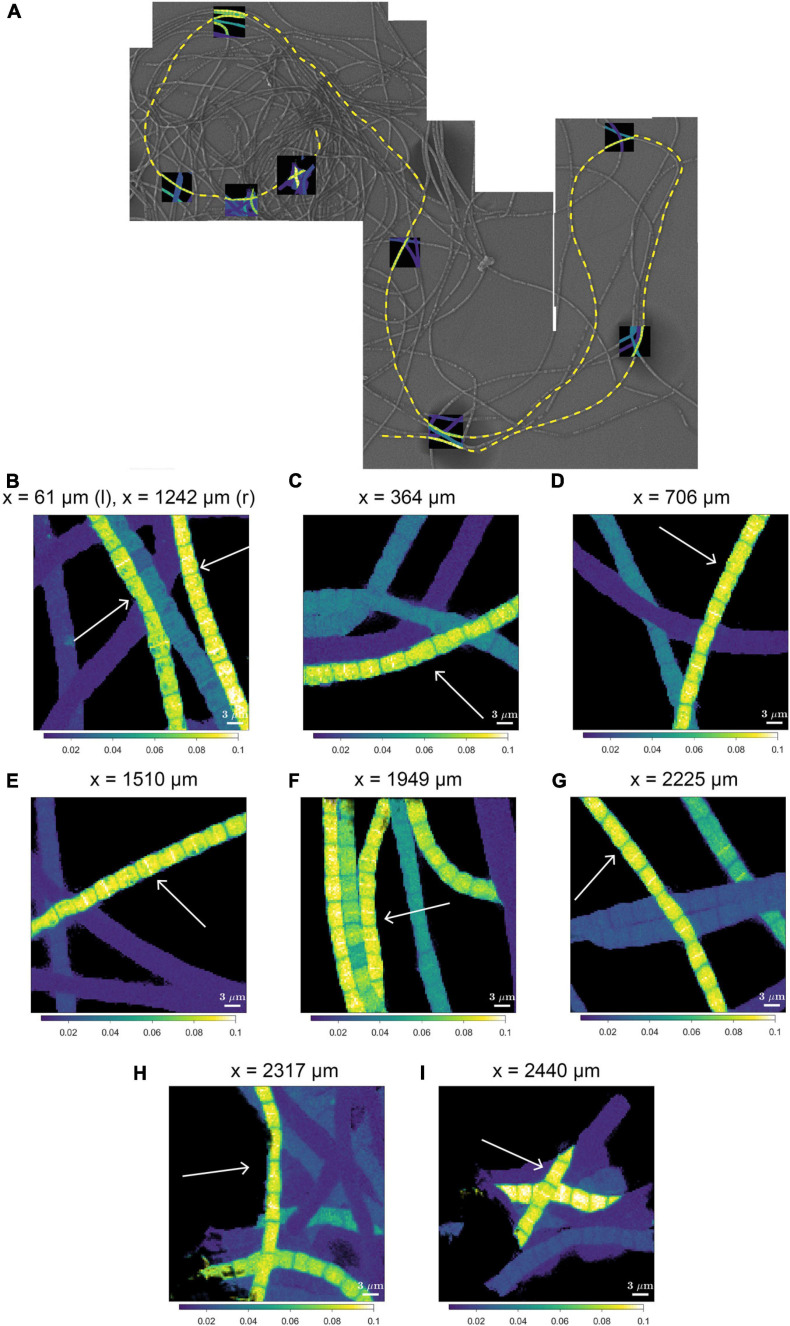
NanoSIMS images of the ^13^C atom fraction measured along the length of a filament in the process of cell division. **(A)** Mosaic image of Scanning Electron Microscopy (SEM) showing a bundle of cable bacterium filaments. The dashed yellow line indicates the filament that was investigated in detail with nanoSIMS (filament #20). NanoSIMS images of the ^13^C atom fraction [calculated as ^13^C/(^12^C + ^13^C)] are superimposed onto the SEM image. The corresponding ^15^N atom fractions can be found in Supplementary Figure 1. **(B–I)** Detailed nanoSIMS images of the ^13^C atom fraction measured in certain segments along the filament. White arrows indicate the segments of the filament that has been analyzed. The distance *x* (as measured from the start of the filament) is indicated on top of each image. Scale bars are all 3 μm. NanoSIMS images of the ^13^C atom fractions of all the 20 other measured filaments can be found in Supplementary Figure 2.

A striking observation by nanoSIMS is that when bands of increased ^13^C and ^15^N atom fractions were present in the mid-plane of cells in highly labeled filaments, these bands were observed in nearly all cells along the length of a filament. This is best illustrated by data from 9 separate segments over a distance of 2.3 mm along a single filament taken from the suboxic zone (#20; Figure 4 and Supplementary Figure 2) and was additionally observed in four more filament, all from the suboxic zone of the sediment (Supplementary Figure 2). No bands with enhanced labeling were observed in any of the other filaments (Supplementary Figure 2) whose average labeling was lower (Figure 1 and Table 1), indicating that this banding pattern is only observed in filaments that experienced a period of fast growth during the labeling period.

We interpret the bands with the locally enhanced ^13^C and ^15^N enrichment as division planes. Our observations thus suggest that cell division in cable bacteria occurs synchronously along the filament in the suboxic part of the filament. Asynchronous division, where some cells are dividing and the rest are not dividing was not observed.

### No Cell Division in Cells in the Oxic Zone

**FIGURE 5:**
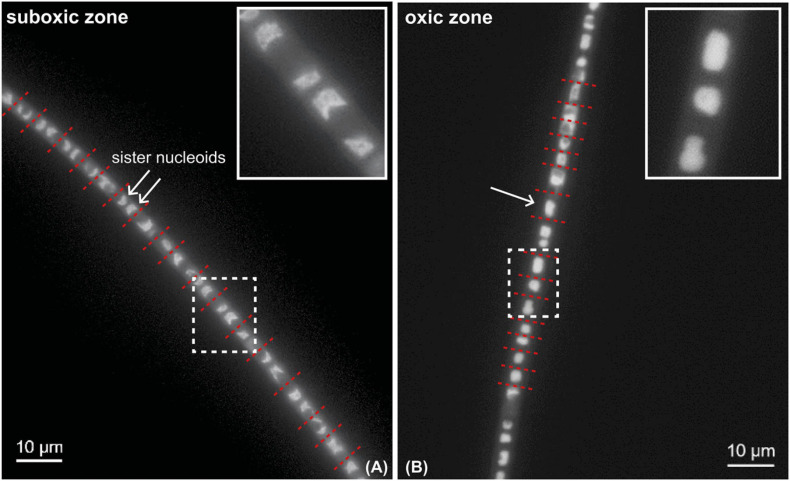
DAPI-stained fluorescence microscopy image of a cable bacterium filament. DAPI stains the DNA inside the cells. White arrows mark individual nucleoids. Red dotted lines show the position of the cell septa. Insets show a close-up of areas indicated by white dotted rectangles. Segments from the same filament are shown, but residing in the oxic and suboxic zones. **(A)** Cells in the suboxic zone show two separated chromosomes in a crescent-shape at each of the cell poles. **(B)** Cells in the oxic zone show a single condensed chromosome that occupies a large part of the cell volume.

Segments of the same filaments were analyzed from both the oxic and suboxic zone. DAPI-staining revealed that consecutive cells in the suboxic zone had separated crescent-shaped sister chromosomes that were located at the poles of the cell (Figure 5A). In contrast, no chromosome separation was observed in the segment that was present in the oxic zone (Figure 5B). The cells located in or close to the oxic zone mostly showed a single condensed chromosome that was either round or showed a cloud-like appearance in the middle of the cell that takes up most of the cell volume (Figure 5B).

### Genes Involved in Cell Division

The recently published genome data of two marine cable bacteria species (*Candidatus* Electrothrix aarhusiensis and *Ca*. Electrothrix marina sp. A5; Kjeldsen et al., 2019) were examined for the genes involved in cell division, and compared to genome data from two closely related sulfate reducers (*D. propionicus* and *D. japonicus).* All four species belong to the family *Desulfobulbaceae* in the class *Desulfobulbia*.

Overall the gene toolbox for cell division in cable bacteria strongly resembles that in *Escherichia coli*, the model Gram-negative microorganism for which the process of cell division has been most intensively studied (Table 2). The genomes of *Ca.* E. aarhusiensis and *Ca.* E. marina contain most genes that constitute the division and cell wall (*dcw*) cluster as well as other genes involved in cell division in *E. coli* (detailed list in Table 2). Differences in gene presence between the two cable bacteria genomes are most likely due to the incompleteness of the draft genome sequences.

**TABLE 2:** Genes putatively involved in cell division present in genomes of cable bacteria and *Desulfobulbus* spp.

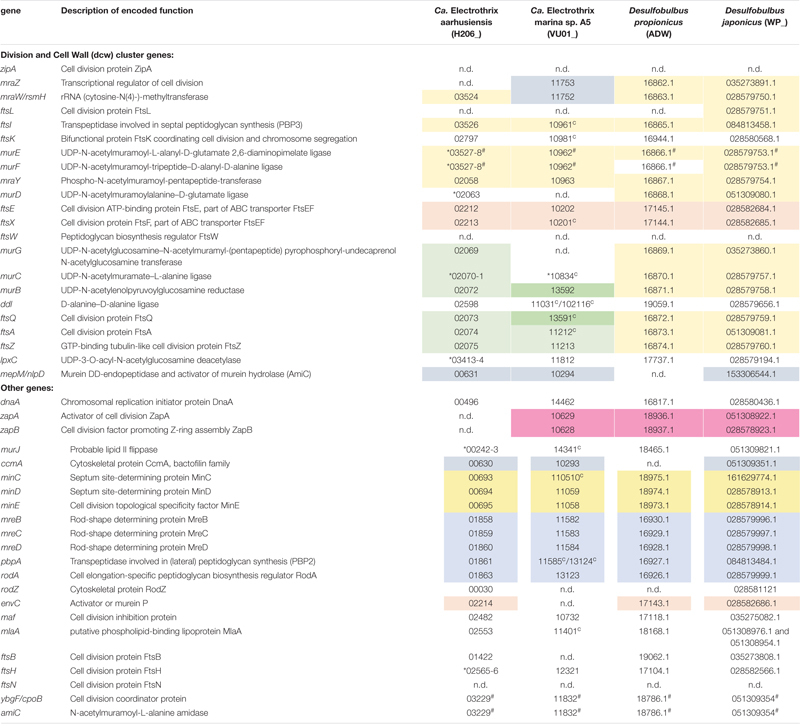

*Indicates the presence of frameshift(s) compared to the gene and its translated protein sequence listed in NCBI with the denoted identifier. ^#^Fused genes. ^c^Genes located at the start or end of a contig, resulting in a partial sequence. n.d., not found in genome. Blocks of the same color denote gene clusters of adjacent genes (including max. 1 gene per cluster putatively encoding a small hypothetical protein). Gene numbering is not necessarily sequential.

With respect to the genes involved in cell division (Table 2), we observed no differences between the filamentous cable bacteria and non-filamentous *D. propionicus* and *D. japonicus*. Comparison between the genome of *E. coli* on the one hand, and the two genomes of *Ca.* Electrothrix and the two *Desulfobulbus* species on the other hand, revealed that *zipA*, *ftsW* and *ftsN* were absent in all four *Desulfobulbaceae* genomes. The gene *ftsL* was detected in the genome of *D. japonicus*, but appears to be absent in many other genomes of the *Desulfobulbaceae* (MacGregor et al., 2017). An analysis across different phyla has shown that *zipA*, *ftsL*, *ftsN* and to a lesser extent *ftsW* are lacking in many bacterial genomes (Margolin, 2000).

In the genomes of the two *Desulfobulbus* species, the composition and synteny of the *dcw* cluster as present in *E. coli* is largely preserved (Table 2, blocks of the same color indicate gene clusters). In the genome of *Ca*. Electrothrix aarhusiensis other genes have been inserted between *mraY*, *murD*, and *murG* compared to the *Desulfobulbus* genomes. We do not know whether the same is true for *Ca.* Electrothrix marina because this genome consists of many relatively short contigs.

## Discussion

**FIGURE 6:**
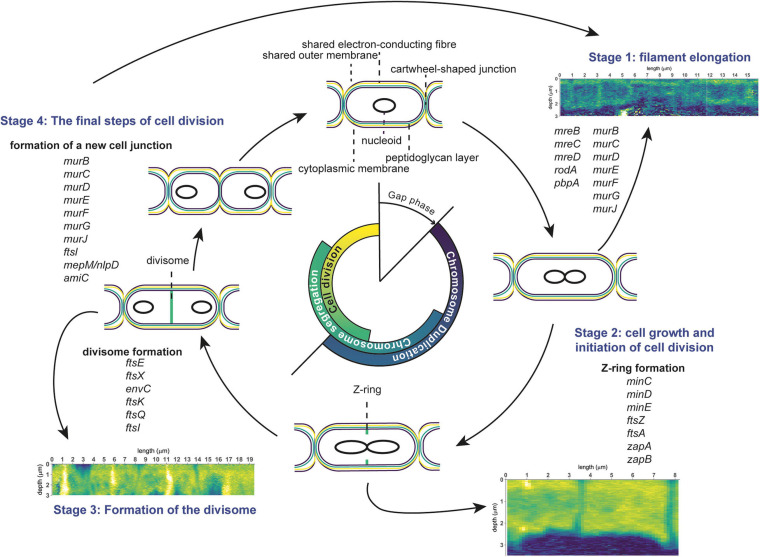
Schematic overview of the cell division cycle in cable bacteria. Cell cycle events are shown, together with the corresponding stages observed in nanoSIMS images (^13^C atom fraction), as well as information from genome analysis of cable bacteria (genes involved in cell division). The three stages represent cell elongation, cell growth and cell division. See text for details.

**FIGURE 7:**
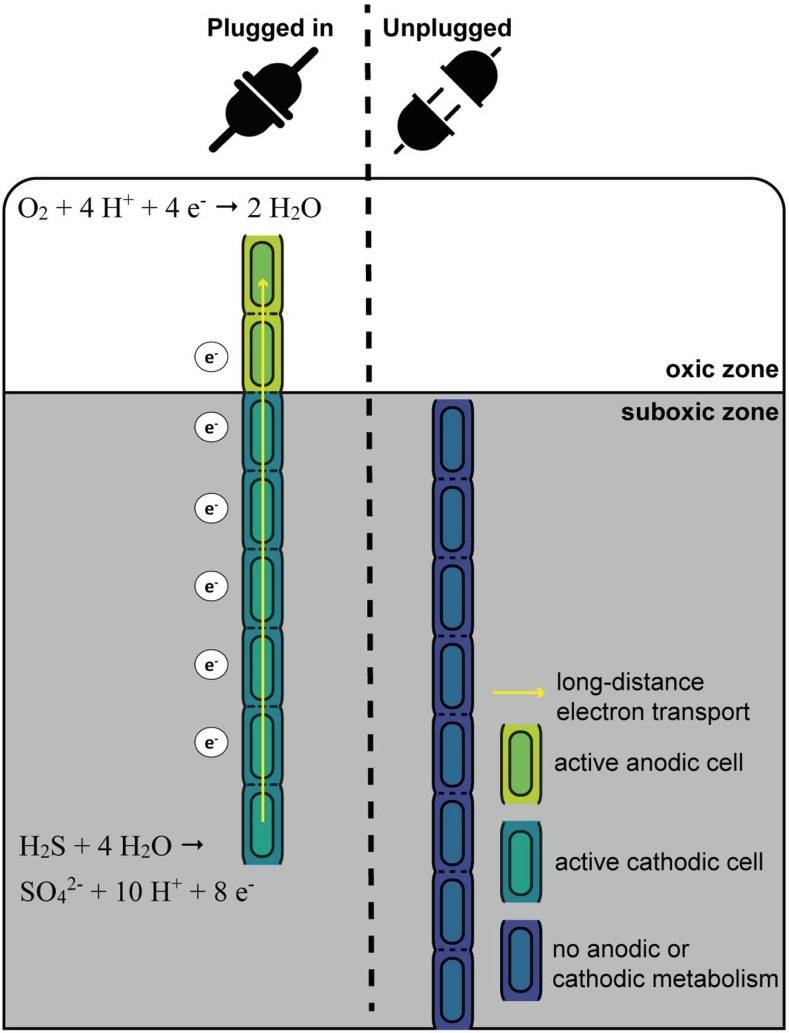
Schematic overview of the “oxygen pacemaker” hypothesis. A filament can be in two states: “plugged in” and “unplugged.” If a filament is “plugged in,” part of it has access to oxygen. Only in this state has the filament the capacity to oxidize sulfide via long-distance electron transport. If a filament is “unplugged,” access to oxygen is lost and the filament no longer has the capacity to perform long-distance electron transport and thus generate energy.

The combined results of our nanoSIMS, fluorescence microscopy and genomic analyses provides insight into the cell cycle (scheme in Figure 6) and growth mechanism (scheme in Figure 7) of cable bacteria. Below we first discuss the cell cycle at the level of a single cell by combining insights from genomic data with nanoSIMS images that capture “snapshots” of major cell cycle events. Genomic data about genes involved in cell division are compared to the Gram-negative model organism *E*. *coli* to assess the differences and similarities. Then, we “zoom out” to discuss the process of growth and cell division at the level of a multicellular filament. Here, we propose a model for the lifestyle of a cable bacterium based on our nanoSIMS analysis and fluorescence microscopy.

### The Cell Cycle of Cable Bacteria

In general, the bacterial cell cycle includes three activities: chromosome replication, chromosome segregation, and cell division (Figure 6; Dewachter et al., 2018). The three activities are not sequential but show an overlap (Figure 6). Specifically, both chromosome segregation and the onset of cell division start before the chromosome is fully replicated. Cellular growth takes place throughout the whole cycle, but biomass synthesis rates vary and are highest during chromosome replication. Most bacteria carefully maintain their size over different generations, therefore, cell growth and progression of the cell cycle must be intimately connected and coordinated (Dewachter et al., 2018). Chromosome replication is dependent on the growth rate, and both the onset and duration are governed by nutrient availability and metabolic status, which hence allows cells to maintain genome integrity in fluctuating nutrient conditions (Wang and Levin, 2009). Cellular growth starts with cell elongation which is followed by septum formation prior to division. Elongation and septum formation do not take place simultaneously and are characterized by the activity of different protein complexes (the elongasome and the divisome, respectively; Scheffers and Pinho, 2005; Egan et al., 2020).

As defined above, the cell envelope of cable bacteria includes the full conductive network and includes both the lateral cell envelope, which harbors the conductive periplasmic fibers, and the cell septa that harbor the conductive cartwheel structure (Cornelissen et al., 2018; Jiang et al., 2018; Meysman et al., 2019; Thiruvallur Eachambadi et al., 2020). Recently, the lateral cell envelope has been shown to consist of the cytoplasmic membrane, a peptidoglycan layer, the electron-conducting fiber network and the outer cell membrane (Boschker et al., 2020). Although our nanoSIMS measurements have a high spatial resolution (lateral: ∼130 nm, depth: ∼30 nm; Figure 2), this resolution is insufficient to discriminate between the different sub-structures of the cell envelope. However, it does allow discrimination of isotope label incorporation between the cell envelope and the cytoplasm. The latter provided useful information of the different stages of the cell division cycle.

Based on this 3D nanoSIMS data, we now attempt to reconstruct the cell cycle of a cable bacterium cell. The reconstruction is guided primarily by the current knowledge of the standard cell cycle for the model organism *E. coli* combined with a comparative analysis of the putative genes involved in cell division. We emphasize, however, that because nanoSIMS analysis lacks molecular specificity, further research that combines methods such as immunolabeling and correlative microscopy is required to verify the identity of the structures and proteins proposed in our model.

#### Stage 1: Cell Elongation

The cell cycle in cable bacteria starts with the elongation of the longitudinal cell envelope (Figure 6, stage 1). NanoSIMS analysis captured some filaments in this stage: they show a labeling pattern where the cell envelope (encompassing both longitudinal cell wall and septa) is more labeled with ^13^C (Figures 2A,B) compared to the cytoplasm (which has a labeling level closer to the natural ^13^C atom fraction). We interpret the observed integration of labeled carbon in the cell envelope during the 6 h incubation as the lateral elongation of the cell envelope and a remnant of cell junction formation that preceded cell elongation. This would imply a relatively short gap phase in this filament. Precursors for cell envelope components are synthesized in the cytoplasm and then transported to the periplasm (Egan et al., 2017, 2020). The cytoplasm constitutes a larger carbon pool than the cell envelope, thus a comparable absolute amount of precursor build-up would lead to a lower ^13^C atom fraction in the cytoplasm (due to the larger initial amount of ^12^C) and a higher ^13^C atom fraction in the cell envelope (as newly synthesized material is concentrated there). This can explain the lower labeling in the cytoplasm.

Elongation of the lateral cell envelope hence must include synthesis of both the lipid membranes and the peptidoglycan sacculus as well as elongation of the electron-conducting fiber structure network. The peptidoglycan layer in Gram-negative bacteria consists of a three-dimensional macromolecular network that surrounds the cytoplasmic membrane. It is very thin and only comprises 1 to 3 sheets of murein consisting of glycan strands cross-linked by peptide-chains. Despite being this thin (max. ∼ 7 nm), the peptidoglycan layer is the principal stress-bearing and shape-maintaining structure, which is of critical importance to cell viability (Höltje, 1998; Scheffers and Pinho, 2005). The fiber network embedded in the periplasm is a conspicuous structure unique to cable bacteria and carries the electron flow that connects the half-reactions of sulfide oxidation and oxygen reduction (Meysman et al., 2019). It consists of a series of 15–60 parallel fibers (depending on filament diameter), which are ∼50 nm in diameter, and run in parallel along the longitudinal axis of the filaments. The fibers are continuous at the cell junctions, but are also sideways connected by a conductive cartwheel structure that converges to a central node, thus providing electrical redundancy (Cornelissen et al., 2018; Thiruvallur Eachambadi et al., 2020). This fiber network is of critical importance to the metabolic functioning of a cable bacterium filament, while it is also believed to contribute to the shape and mechanical integrity of the filament (Cornelissen et al., 2018; Jiang et al., 2018). Therefore, the lateral insertion of new material in the cell envelope, whether this happens in the peptidoglycan layer or the conductive fibers, must be tightly controlled to ensure viability of both the cell and the filament. Our nanoSIMS data (Figures 2A,B) indicate that the elongation takes place all along the whole lateral cell envelope, and hence not at a single point. This suggests that lipid membranes, the peptidoglycan layer and the conductive fibers are continuously and homogeneously elongated in different places. Intriguingly, it appears that elongation takes place while the filament is metabolically active, i.e., while there is long-distance electron transport in the conductive fiber network. Recent research using high-resolution atomic force microscopy showed that electrical currents can still flow along the filament even if part of the fiber structure is disconnected (i.e., some fibers are no longer continuous) (Thiruvallur Eachambadi et al., 2020). Thus, it appears that cells can elongate while still carrying current as long as the network of fibers surrounding them do not elongate all at the same time.

The genes responsible for construction of the conductive fibers remain presently unknown (Kjeldsen et al., 2019), and so genome analysis cannot provide information about the proteins involved in fiber elongation and synthesis. In contrast, substantial information is available about the biochemistry of peptidoglycan synthesis. Proteins involved in peptidoglycan synthesis are conserved across the bacterial domain, and the corresponding genes are also present in the genomes of *Ca.* Electrothrix aarhusiensis and *Ca.* E. marina. Based on the similarity of the genetic information, it appears that the mechanisms used to elongate the cell envelope and synthesize peptidoglycan in cable bacteria are similar to those employed by other Gram-negative rod-shaped bacteria. In general, lateral peptidoglycan synthesis is aided by a cytoskeleton built by the MreBCD protein complex. MreB is a bacterial homolog of actin that determines the cell shape and guides the synthesis of lateral peptidoglycan, which in itself is catalyzed by a peptidoglycan synthase (penicillin-binding protein PBP2) and RodA. Absence of one (or more) of the Mre proteins or RodA leads to drastic changes in cell morphology and cell viability in *E. coli* (Kruse et al., 2005; Schoenemann and Margolin, 2017). However, these genes are all present in both *Ca.* Electrothrix and both *Desulfobulbus* spp., and in fact, the genes that encode for the Mre complex, PBP2 and RodA (*mreABC*, *pbpA*, and *rodA*) are located in a single gene cluster (Table 2). The genes encoding the enzymes to synthesize peptidoglycan precursors as identified in *E. coli* (MurB to MurG, and MurJ) are also present in the two cable bacteria and *Desulfobulbus* species analyzed (Table 2). The activity of MurB, followed by the activity of MurC, MurD, MurE, and MurF in the cytoplasm creates Lipid I, a membrane-bound peptidoglycan precursor. MurG is an essential transferase operating at the cytoplasmic membrane that transforms Lipid I to Lipid II, the basic building block of peptidoglycan synthesis. The lipid II can then be transferred to the periplasm by the flippase MurJ, where it is cross-linked to form new peptidoglycan by cross-linking of the stem-peptides, which is catalyzed by peptidoglycan synthases (penicillin binding proteins) (Egan et al., 2017, 2020).

#### Stage 2: Growth and Initiation of Cell Division

For a successful cell division, chromosome duplication must be coordinated with accurate segregation of the newly replicated chromosomes, and also with cell growth and division (Reyes-Lamothe et al., 2012). The initiation of replication is dependent on the growth conditions, but it is only partly understood how replication initiation is coordinated with cellular metabolism and growth (Haeusser and Levin, 2008; Reyes-Lamothe et al., 2012; Harris and Theriot, 2016; Reyes-Lamothe and Sherratt, 2019). To date, all bacterial chromosomes have a single replication origin (*oriC*) from where replication, initiated by DnaA, proceeds bidirectionally. We observed the sister chromosomes as crescent-shaped features at the cell poles in DAPI-stained cells (only seen in cells retrieved from the suboxic zone; Figure 5A). The duplicated chromosomes are completely segregated to both cell poles and leave enough space for the divisome and the new septum to be built. The positioning of the segregated chromosomes (nucleoids) is linked to cell division through nucleoid occlusion factors, a defense mechanism made up of proteins that prevent formation of the divisome until chromosome segregation is completed (Reyes-Lamothe and Sherratt, 2019). Precise chromosome positioning and segregation may even (partly) determine when the divisome is built, which is to avoid DNA cleaving during cell division (Reyes-Lamothe et al., 2012).

In our nanoSIMS data, the growth phase is characterized by a relatively homogeneous increase in the ^13^C and ^15^N labeling of the cytoplasm, as observed in filaments displaying medium label uptake (Figures 2D–H, 6, stage 2). Chromosome replication and the subsequent build-up of the divisome requires a substantial amount of biosynthesis (Reyes-Lamothe and Sherratt, 2019), which could explain the increased labeling of the cytoplasm. At this point in the cell cycle, bio-structures in the cell envelope are recycled as much as possible (Park and Uehara, 2008), which can explain why the cell envelope is less enriched in both ^13^C and ^15^N than the cytoplasm. This enrichment pattern was only observed for filaments that were sufficiently active (i.e., acquired sufficiently high amounts of external C an N in the cytoplasm during the labeling experiment). Given the large volume of the cytoplasm, this suggests that high rates of biosynthesis occur over a short time span within this specific part of the cell cycle. This observation is consistent with observations on cable bacterium filaments analyzed with atomic force microscopy where swelling in the middle of cells was observed (Jiang et al., 2018). This swelling was interpreted as a volume increase of cells to accommodate the newly synthesized DNA at the start of chromosome replication (Jiang et al., 2018).

After chromosome replication and subsequent segregation of the sister chromosomes, cell division in *E. coli* is initiated with the assembly of a circumferential scaffold on the cytoplasmic membrane, the Z-ring. The Z-ring is composed of polymerized FtsZ (the prokaryotic homolog of tubulin) and anchored to the inside of the cytoplasmic membrane (Goehring and Beckwith, 2005). The *ftsZ* gene is present in both cable bacteria and *Desulfobulbus* spp. (Table 2). Mid-cell accumulation of FtsZ in cable bacteria has been observed previously using FtsZ-specific immunolabeling in combination with fluorescence microscopy (Jiang et al., 2018). We interpret the ^13^C-enriched circumferential ring observed mid-cell near the cell envelope as a Z-ring (Figures 2E,F). Our short-term labeling experiment shows that the carbon utilized for the build-up of the ring with increased ^13^C atom fraction (which is interpreted as the Z-ring) was newly assimilated during the labeling period (<6 h).

As known from other studies, the formation of the Z-ring is under tight spatial and temporal control to ensure that it is assembled between segregated chromosomes (Haeusser and Levin, 2008; Haeusser and Margolin, 2016; Dewachter et al., 2018). The spatial regulation is controlled by the Min system (MinCDE), for which the genes are present in cable bacteria (Table 2). MinC and MinD form negatively acting gradients that inhibit the activity of FtsZ at the cell poles, while MinE ensures that polymerization of FtsZ takes place at the DNA-free midcell (Haeusser and Levin, 2008; Haeusser and Margolin, 2016; Dewachter et al., 2018). FtsZ does not have affinity for the lipid membrane, so a membrane-tethering protein is required to connect FtsZ to the cytoplasmic face of the inner membrane. In *E. coli* this role is performed by FtsA and ZipA, two proteins that both independently interact with FtsZ to attach it to the membrane (Pichoff and Lutkenhaus, 2002). Cable bacteria lack *zipA*, like bacteria from many other phyla (Margolin, 2000), but do possess *ftsA*, which is present adjacent to *fts*Z in the genome (Table 2). Either ZipA or FtsA is essential for formation and stabilization of the Z-ring in *E. coli* (Pichoff and Lutkenhaus, 2002) and it appears that FtsA fulfils this role in cable bacteria and *Desulfobulbus* spp.

The Z-ring can be further stabilized by interaction with ZapA and ZapB, two small non-essential proteins that interact with FtsZ and are recruited to the Z-ring early in the formation of the divisome (Adams and Errington, 2009). The *zapAB* genes are present in the genome of *Ca.* Electrothrix marina sp. A5 and *Desulfobulbus* spp., but were not found in *Ca.* E. aarhusiensis, likely because of the incompleteness of the latter genome (Table 2).

#### Stage 3: Formation of the Divisome

Once the Z-ring is established, the remaining cell division proteins are recruited onto the Z-ring to form the divisome. There can be a considerable time lag between Z-ring formation and the formation of the divisome (Adams and Errington, 2009; Lutkenhaus et al., 2012; Typas et al., 2012; Dewachter et al., 2018). The completed divisome spans the cell membrane. Our nanoSIMS imaging showed a disk-like structure located in the middle of the cell and characterized by elevated ^13^C and ^15^N atom fractions compared with the rest of the cytoplasm (Figure 3). We interpret this disk as the completed divisome. Because the average ^13^C atom fraction (0.100 ± 0.002) of this disk is close that of the porewater DIC pool (0.112), this suggests that the disk was completely synthesized during the SIP experiment (<24 h) using mostly newly fixed C. The ^15^N atom fraction of the pore water NH_4_^+^ was not measured, but the ^13^C/^15^N ratio in the disk is the same as in the cytoplasm, suggesting that N required for its synthesis also originated externally. This is in contrast to the divisome formation in the filamentous cyanobacterium *Anabaena oscillarioides*, where only N is fixed recently (<4 h), whereas C in the division proteins is derived from internally recycled carbon pools (Popa et al., 2007).

Studies have shown that the *E. coli* divisome contains 11 “late-division” proteins that are assembled to the divisome (FtsEX, EnvC, FtsK, FtsQ, FtsL, FtsB, FtsW, FtsI, FtsN, and AmiC), most of them non-essential (Goehring and Beckwith, 2005; Lutkenhaus et al., 2012). Not all genes coding for these proteins are present in cable bacteria and *Desulfobulbus* spp. (FtsL, FtsW, and FtsN are not encoded; Table 2). It is presently unknown how the division proteins are connected, but FtsA appears to plays a key role (Lutkenhaus et al., 2012; Haeusser and Margolin, 2016). Similar to divisome formation in *E. coli* (Goehring and Beckwith, 2005; Lutkenhaus et al., 2012), the FtsEX complex and its interaction partner EnvC are presumably recruited first. FtsEX is a conserved membrane protein complex that helps in the recruitment of late divisome proteins and aids in the coordination of cell wall hydrolysis when constriction is progressing (Yang et al., 2011; Du et al., 2016; Haeusser and Margolin, 2016). After FtsEX and EnvC, FtsK, FtsQ, and FtsI are recruited to the division plane to complete the formation of the divisome (Lutkenhaus et al., 2012), although a number of other unknown proteins may be involved as well.

#### Stage 4: The Final Steps of Cell Division

In the final step of the cell cycle in *E. coli*, the divisome is activated to synthesize septal peptidoglycan by FtsI activity and the rest of the cell envelope (Lutkenhaus et al., 2012). The driving force behind bacterial fission is the result of membrane constriction applied by the treadmilling of FtsZ filaments, the force applied by the inward growth due to local peptidoglycan synthesis, or a combination of these two processes. In both scenarios, synthesis of the new cell envelope is the rate-limiting step (Coltharp et al., 2016). In single-celled bacteria, the daughter cells separate completely due to amidase activity splitting the septal murein, accompanied by invagination of the outer membrane (Heidrich et al., 2001). In *E. coli*, activity of one of the amidases AmiA, AmiB, or AmiC ensures cell separation (Lutkenhaus et al., 2012). The gene encoding AmiC is present in cable bacteria, as is the gene encoding for its activator NlpD. Although FtsN is required for localization of AmiC in *E. coli* (Lutkenhaus et al., 2012), the gene *ftsN* is absent in many bacteria, including cable bacteria.

In filamentous cable bacteria the outer membrane invaginates slightly at the cell septum but cells do not split completely. For *Desulfurivibrio* strain 1MN, a strain closely related to groundwater cable bacteria (based on the 16S rRNA gene sequence), both single cells and filaments were present in the same culture (Müller et al., 2020), suggesting that the cells have the capacity to divide completely but that this capacity can be suppressed by regulatory control. Besides synthesizing the cytoplasmic membrane and the peptidoglycan layer, cable bacteria also need to synthesize the complex cartwheel structure at the new junction, which becomes part of the conductive fiber network and ensures a fail-safe electrical connection between the newly formed daughter cells (Thiruvallur Eachambadi et al., 2020). The build-up of this cartwheel structure during cell division was previously visualized via focused ion beam scanning combined with scanning electron microscopy (FIB-SEM) (Figure 3D in Cornelissen et al., 2018), and it was hypothesized that it is formed following an invagination of the outer envelope during cell division. FIB-SEM images show that the cartwheel structure starts growing from the outside (i.e., near the fiber network) and steadily grows inward, until the radial cartwheel spokes connect at a central node. The genes involved in the synthesis of the cartwheel structure are presently unknown and might be unique to cable bacteria.

Together, the identification of putative genes involved in cell division and the presence of a ring and a disk with elevated ^13^C and ^15^N values compellingly suggests the presence of a Z-ring and divisome in these cells. However, these cellular features cannot be unequivocally identified by NanoSIMS alone, and so further experimental verification is needed to demonstrate that Z-ring and divisome proteins truly appear during the different stages of the cell division.

### Filamentous Growth of Cable Bacteria

Our SIP-nanoSIMS results provide insight into various aspects of filament growth in cable bacteria.

#### Filamentous Growth of Cable Bacteria Is Non-apical

Previous studies have shown that the growth of cable bacteria is too rapid to be exclusively apical (Schauer et al., 2014). Indeed, cable bacteria can grow to centimeter long filaments in just a few days (Schauer et al., 2014; Vasquez-Cardenas et al., 2015), which requires that the majority of cells within the filament must divide. Previously, continuous (non-apical) division of cells along the length of a filament has been observed over a length of several cells up to a distance of 80 μm (Schauer et al., 2014; Jiang et al., 2018). Here, we show that non-apical filament elongation extends over far larger distances (up to 2.3 mm).

#### Filamentous Growth Only Takes Place in the Suboxic Zone

Based on genome analysis (Kjeldsen et al., 2019) and nanoSIMS tracking of ^13^C and ^15^N assimilation (Geerlings et al., 2020), it has been hypothesized that cell division occurs along the anodic part of the filament (sulfide-oxidizing cells located in the suboxic zone of the sediment), but not along the cathodic part of the filament (oxygen-reducing cells located in the oxic zone). Since ∼90% of the filaments is estimated to be present in the suboxic zone of the sediment, this would imply that ∼90% of the cells could potentially contribute to filament growth.

Our data provide multiple lines of evidence that cell division only occurs in the suboxic zone. Firstly, little labeling was observed in the three filaments retrieved from the oxic zone (Figure 1). This is in agreement with a previous SIP experiment where little or no uptake of carbon and ammonia was observed in filaments retrieved from the oxic zone (Geerlings et al., 2020). Secondly, we performed fluorescence imaging of a DAPI-stained filament that spanned both the oxic and suboxic zones (Figure 5). Cathodic cells that were present in or close to the oxic zone possessed a single nucleoid (region containing DNA), which was spherical or oval shaped or showed a cloud-like appearance and was located in the middle of the cell (Figure 5B). In contrast, anodic cells located in the suboxic zone showed two separated nucleoids, which were crescent shaped and located at the cell poles (Figure 5A). Accordingly, while most cathodic oxic cells showed no sign of cell division, the anodic cells of the same filaments were in the process of chromosome segregation.

Our observations hence confirm that cable bacteria display an intriguing division of labor between cells of the same filament where cathodic cells residing in the oxic zone cannot generate energy and therefore cannot assimilate carbon to grow and divide, whereas anodic cells in the suboxic zone do have the capacity to grow and divide (Kjeldsen et al., 2019; Geerlings et al., 2020).

#### The “Oxygen Pacemaker” Hypothesis

One conspicuous observation is that cells within a given filament are homogenously labeled, while large differences are observed in the degree of labeling among different filaments retrieved from the same sediment (small within-filament variation, large between-filament variation; Figure 1, Geerlings et al., 2020). These data provide a number of insights, which we synthetize here as the “oxygen pacemaker” hypothesis. This idea rests upon the following premises (summarized in Figure 7):

1. Energy conservation (ATP formation) and biomass synthesis are only linked to anodic sulfide oxidation.
2. Biomass synthesis within a cell only occurs when the filament performs long-distance electron transport.
3. A filament can only perform long-distance electron transport when one part of the filament has access to oxygen.
4. Filaments regularly disconnect from oxygen (i.e., retract into the suboxic zone).

The exclusive conservation of energy during anodic sulfide oxidation has already been discussed in depth above (section “Filamentous Growth Only Takes Place in the Suboxic Zone”). Equally, the observation that cells within a single filament show a similar degree of labeling suggest that label uptake occurs in a synchronized fashion across millimeter long filaments. We propose that such synchronization occurs through long-distance electron transport, which is a process that instantaneously and simultaneously can affect all cells within a given filament. All cells within a cable bacterium filament have the capacity for oxygen reduction and immediately use this capacity once exposed to oxygen (Geerlings et al., 2020). Growth and cell division in a filament can only take place when there are both active anodic and cathodic cells. Without access to oxygen, electron transport in a filament is immediately halted (Bjerg et al., 2018), as the periplasmic wire network becomes saturated with electrons (Meysman et al., 2015). This would stop all endergonic catabolic activity, and hence, ATP production and biosynthesis would seize. Once a filament has access to oxygen, electrons are removed from the periplasmic wire network, and model simulations suggest that this causes a potential drop that is quite similar along the whole anodic part of a filament (Meysman et al., 2015). This could be the sought-after signal for all sulfide-oxidizing cells within a filament to start carbon and nitrogen assimilation.

Effectively, with respect to oxygen access, one can imagine a filament to be in two states (Figure 7): “plugged in” (where part of the filament resides in the oxic zone) and “unplugged” (where the entire filament resides in the anoxic zone). Filaments can only perform long-distance electron transport when they are “plugged in”, as only in this state can the filament release electrons from the internal conductive network through oxygen reduction by the cathodic cells that reside in the oxic zone. In contrast, when a filament is “unplugged”, the internal conductive network is rapidly saturated with electrons, and so additional electrons can no longer be “uploaded” to the network leading to a halt of the anodic sulfide oxidation. Critically, the status of being “plugged in” or “unplugged” is immediately felt by all cells in the filament, and this explains the homogeneous label uptake across filaments. Additionally, the fact that we see strong variation in label uptake among filaments then implies that filaments have been in the “plugged in” state for different amounts of time during our 6 and 24 h labeling experiments. In other words, it implies that different filaments had access to oxygen for different periods of time. Hence, the contact with oxygen serves as a “pacemaker” for long-distance electron transport, as well as energy conservation and biomass synthesis in anodic cells.

#### Synchronized Cell Division Within Cable Bacteria

In addition to homogeneous label uptake, filaments display an even more conspicuous form of synchronization. When we examined different segments, spatially segregated along a 2.3 mm long filament stretch, we found that all investigated cells had a similar labeling pattern with a highly enriched band in the middle of each cell (Figure 4). This implies that all 774 cells in this filament (assuming a mean cell length of 3 μm) were in exactly the same phase of cell division (i.e., stage 3, divisome formation) when this filament was retrieved after 24 h and had acquired label during the time it was “plugged in.” This suggests that cell division occurs in a synchronized way over large distances within cable bacterium filaments. These findings are strengthened by the observation of four other synchronously dividing filaments (Supplementary Figure 2). Foremost, we do not think that synchronization is a remnant of the initiation of enrichment culturing. Our sediment enrichment cultures developed an active cable bacteria population during several weeks (pre-labeling times of 26 and 54 days) before addition of labeled substrates. If cell division were initially synchronous in a filament, one would expect such synchronicity to be lost over the incubation period of 26–54 days. However, this was not what we observed: cells within the measured filaments showed synchronous growth, and if a cell cycle phase could be determined, all measured cells in a filament were in the same phase of the cell cycle (Figures 1, 4 and Supplementary Figure 2).

Other instances of synchronous cell division can be found in nature. Early development in animals is characterized by rapid synchronous cell divisions (Satoh, 1977; Newport and Kirschner, 1982). However, synchronicity is quickly lost after five cell divisions. In the bacterial realm, it is also possible to produce synchronously dividing single-celled cultures by enforcing a stationary phase where cells are first starved and none of the cells undergo division, and then induce cell divisions by supplying new medium to the culture. So here synchronized cell division is triggered by an external cue (substrate availability). After several subsequent rounds of cell division, the synchrony is lost and cells are dividing asynchronously within the culture (Cutler and Evans, 1966). Cultures of unicellular *Prochlorococcus* and *Synechococcus* cyanobacteria can maintain a highly synchronized cell cycle which was attributed to a diel expression pattern of the *dnaA* and *ftsZ* genes involved in DNA replication and cell division, respectively. The timing of external light/dark conditions continuously (re)sets and adjusts the pace of cell division and thus maintains cell synchrony in these phototrophic cyanobacteria (Holtzendorff et al., 2001; Asato, 2003). Again synchronized cell division is triggered by an external environmental cue (light/dark cycle). In general, synchronization in a microbial culture is achieved through an external “reset” of the cell cycle, where all cells “sense” a (sudden) change in environmental conditions, i.e., access to energy. This allows for temporally synchronized gene expression and synchronization of the cell cycle.

The synchronized cell division as seen in cable bacteria appears to be rare in other filamentous bacteria. In filamentous cyanobacteria, cell division is generally spread throughout the filament (i.e., not apical), but it is not synchronized. The marine filamentous non-heterocystous diazotrophic cyanobacterium *Trichodesmium erythraeum* IMS101 did not showed synchronized cell division within a filament when grown under controlled nitrogen-fixing conditions (Sandh et al., 2009). Cell division was restricted to small groups of cells that were spread along the filament, and at any given point in time the proportion of dividing cells within a filament varied between 5 and 20% (Sandh et al., 2009). A similar result was observed for the filamentous heterocystous cyanobacterium *Anabaena oscillarioides*, where uptake of ^13^C-labeled bicarbonate over a length of 50 cells was relatively uniform among the vegetative cells performing photosynthetic C fixation, but much lower in the N_2_-fixing heterocysts. Interestingly, the authors observed a cell division plane, but only in one cell (Popa et al., 2007), implying that cell division within *A. oscillarioides* was not synchronized. The filamentous *Anabaena* sp. PCC 7120, a model organism mainly used to investigate the spatial patterning of the nitrogen-fixing heterocysts, showed a strong bias toward even-numbered intervals of vegetative cells between terminally differentiated heterocysts. This bias was attributed to synchronous division of the vegetative cells during the formation of mature heterocysts, which is externally controlled by nitrogen deprivation (Khudyakov and Golden, 2004). Quantitative mathematical modeling performed to understand controls on heterocyst formation within *Anabaena* filaments (Muñoz-García and Ares, 2016) was able to reproduce the observed fraction of even-numbered intervals when stochastic noise in the duration of the cell cycle was kept low (i.e., synchronous cell division). In contrast, high levels of stochastic noise (i.e., unsynchronized cell division) resulted in a percentage of even-numbered intervals close to 50% (Muñoz-García and Ares, 2016). This suggests that when the regulation of cell division is governed by an external cue (e.g., nitrogen deprivation) it can lead to synchronized cell division. However, if the external control disappears, even a small variance in the division time will eventually result in an asynchronously dividing population (Olejarz et al., 2018).

For all cells within the sulfide-oxidizing part of a cable bacterium filament to divide at the same time, there needs to be a “reset” that applies collectively to all cells within a filament. As discussed above, this “common reset signal” is most likely access to oxygen. When a filament gains access to oxygen, synchronized cell growth is initiated, which then can lead to synchronized cell division among the anodic cells within a filament. However, this synchrony would be lost after multiple rounds of cell division. Since asynchronous cell division is not observed and carbon and nitrogen assimilation among cells within a filament is synchronous, filaments must regularly lose access to oxygen and then “reconnect”, at least once every few cell cycles to retain the “metronome effect”. Cable bacterium filaments possess gliding motility and oxygen chemotaxis, and have been seen to frequently reposition themselves in the sediment (Bjerg et al., 2016). Therefore, one option is that filaments, or at least parts of them, frequently migrate in and out of the oxic zone. Because the oxygen-reducing cells in a filament do not have the capacity for energy generation and are subjected to oxidative stress (Kjeldsen et al., 2019), these cells deplete their energy reserves (e.g., polyphosphates) and stop growing for as long as they reside in the oxic zone (Geerlings et al., 2020). Therefore, these oxygen-reducing cells either die or must retreat back into the suboxic zone (Geerlings et al., 2020). Either way, “new” oxygen-reducing cells need to position themselves into the oxic zone to ensure the electrical connection between the two catabolic half-reactions. Until this happens, the metabolism of a filament is paused. Once a satisfactory access to oxygen is re-established, cells residing in the suboxic zone can (re-)start their cell cycle, which will result in another round of synchronized cell division. Differences in access to oxygen and the time necessary to re-establish this connection could explain both the low intra-filament variation, which includes the synchronized cell division, and the high inter-filament variation in C and N assimilation by cable bacteria.

### Summary and Outlook

In conclusion, our data sheds light on the cell cycle in cable bacteria (Figure 6), as well as the mechanism of filament growth (Figure 7). Our data reveals that the cell cycle of cable bacteria is highly similar to that of the Gram-negative model bacterium *E. coli*. Moreover, when comparing cable bacteria to single-celled *Desulfobulbus* species, there is no observable difference in genome content with respect to genes involved in cell division. However, there must be some modifications in the cell cycle to account for the multicellular nature of cable bacteria and the build-up of the electron-conducting fiber network and the cartwheel structure that enables long-range conduction. The mechanism for being filamentous is likely under regulatory control, whereas the genes responsible for being electrogenic (conductive structures) remain unresolved.

Our nanoSIMS data show that carbon (and nitrogen) assimilation in cable bacteria filaments is homogeneous, confirming that growth is distributed along the filament, and is not just apical. Additionally, our data confirm previous observations that growth is restricted to anodic cells residing in the suboxic zone.

Synchronized cell division can be achieved if cells are collectively “starved” and then collectively restart their metabolism. The most likely hypothesis for the observed synchronous growth and division in cable bacteria is that filaments lose their access to oxygen on a regular basis (or at least once per cell cycle). Due to the shared responsibility for energy generation among cells within a filament, losing the connection to oxygen results in a sudden halt of the metabolism in all cells. Regaining access to oxygen restarts the metabolism allowing all cells in the sulfide-oxidizing part of the filament to start growing and eventually divide synchronously.

Future research on cell division in cable bacteria should aim to observe the development of individual filaments in real time and include molecular approaches to monitor gene expression or protein synthesis at the level of individual cells within a filament. Therefore new methods need to be developed where individual filaments can be labeled and the position of cells along a filament can be traced back to either the suboxic or the oxic zone. Distinct processes in the first and last steps of the cell cycle, needed to produce a cell capable of electron transport, are still to be elucidated. New experimental approaches need to be developed to selectively monitor/modify environmental conditions along specific parts of a filament such that the growth can be quantified over a filament stretch that contains both the cathodic and anodic cells.

## Data Availability Statement

The raw data supporting the conclusions of this article are made available in the Supplementary Datasheet 1 provided in the Supplementary Material.

## Author Contributions

NG, FM, HB, JM, and LP conceived the study. NG and SH-M set up the enrichment culture and prepared all samples for nanoSIMS analysis. JG performed the genomic analysis. DV-C did the DAPI staining and fluorescence microscopy analyses. MK performed the nanoSIMS analysis. NG, MK, and LP analyzed the nanoSIMS data. NG wrote the manuscript with contributions from all co-authors. All authors contributed to the article and approved the submitted version.

## Conflict of Interest

The authors declare that the research was conducted in the absence of any commercial or financial relationships that could be construed as a potential conflict of interest.

## Footnotes

1 ftp.ncbi.nlm.nih.gov/genomes/genbank/bacteria/

## Supplementary Material

Supplementary Figure 1 NanoSIMS images of the ^15^N atom fraction measured along the length of a filament in the process of division. The corresponding SEM image and the nanoSIMS images of the ^13^C atom fractions are shown in Figure 4.

Supplementary Figure 2 NanoSIMS images of the ^13^C atom fraction measured along the length of all followed filaments. Mosaic image obtained via scanning electron microscopy (SEM) showing the filaments measured, as indicated by dashed lines.

Supplementary Method Stepwise description of how to visualize the depth variation in the nanoSIMS data along a lateral profile using Look@NanoSIMS.

Supplementary Datasheet 1 Raw data showing ^13^C and ^15^N atom fractions in individual cells from the filaments analyzed in this study.
