# Supplementary material for: Cell Cycle, Filament Growth and Synchronized Cell Division in Multicellular Cable Bacteria
Source: Front Microbiol. 2021 Jan 27;12:620807. doi: 10.3389/fmicb.2021.620807 (PMC7873302; doi:10.3389/fmicb.2021.620807)
Supplement: Supplementary Figure 1 — NanoSIMS images of the 15N atom fraction measured along the length of a filament in the process of division. The corresponding SEM image and the nanoSIMS images of the 13C atom fractions are shown in Figure 4. [file Data_Sheet_2.PDF]

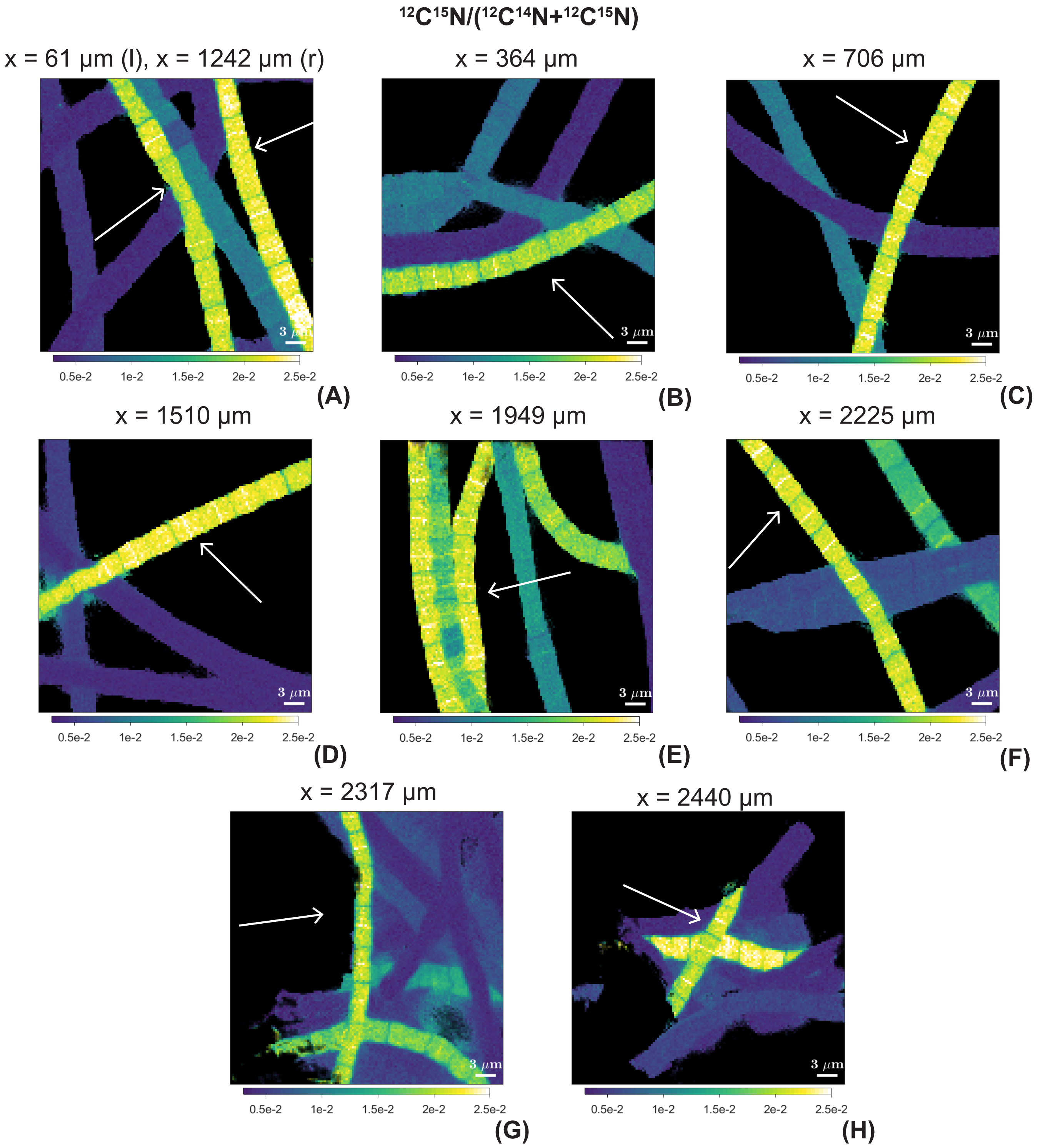

$^{12}C^{15}N/(^{12}C^{14}N+^{12}C^{15}N)$
x = 61 μm (l), x = 1242 μm (r)
x = 364 μm
x = 706 μm
x = 1510 μm
x = 1949 μm
x = 2225 μm
x = 2317 μm
x = 2440 μm
3 μm
0.5e-2
1e-2
1.5e-2
2e-2
2.5e-2
(A)
(B)
(C)
(D)
(E)
(F)
(G)
(H)
