# Supplementary material for: Cell Cycle, Filament Growth and Synchronized Cell Division in Multicellular Cable Bacteria
Source: Front Microbiol. 2021 Jan 27;12:620807. doi: 10.3389/fmicb.2021.620807 (PMC7873302; doi:10.3389/fmicb.2021.620807)
Supplement: Supplementary Figure 2 — NanoSIMS images of the 13C atom fraction measured along the length of all followed filaments. Mosaic image obtained via scanning electron microscopy (SEM) showing the filaments measured, as indicated by dashed lines. [file Data_Sheet_3.PDF]

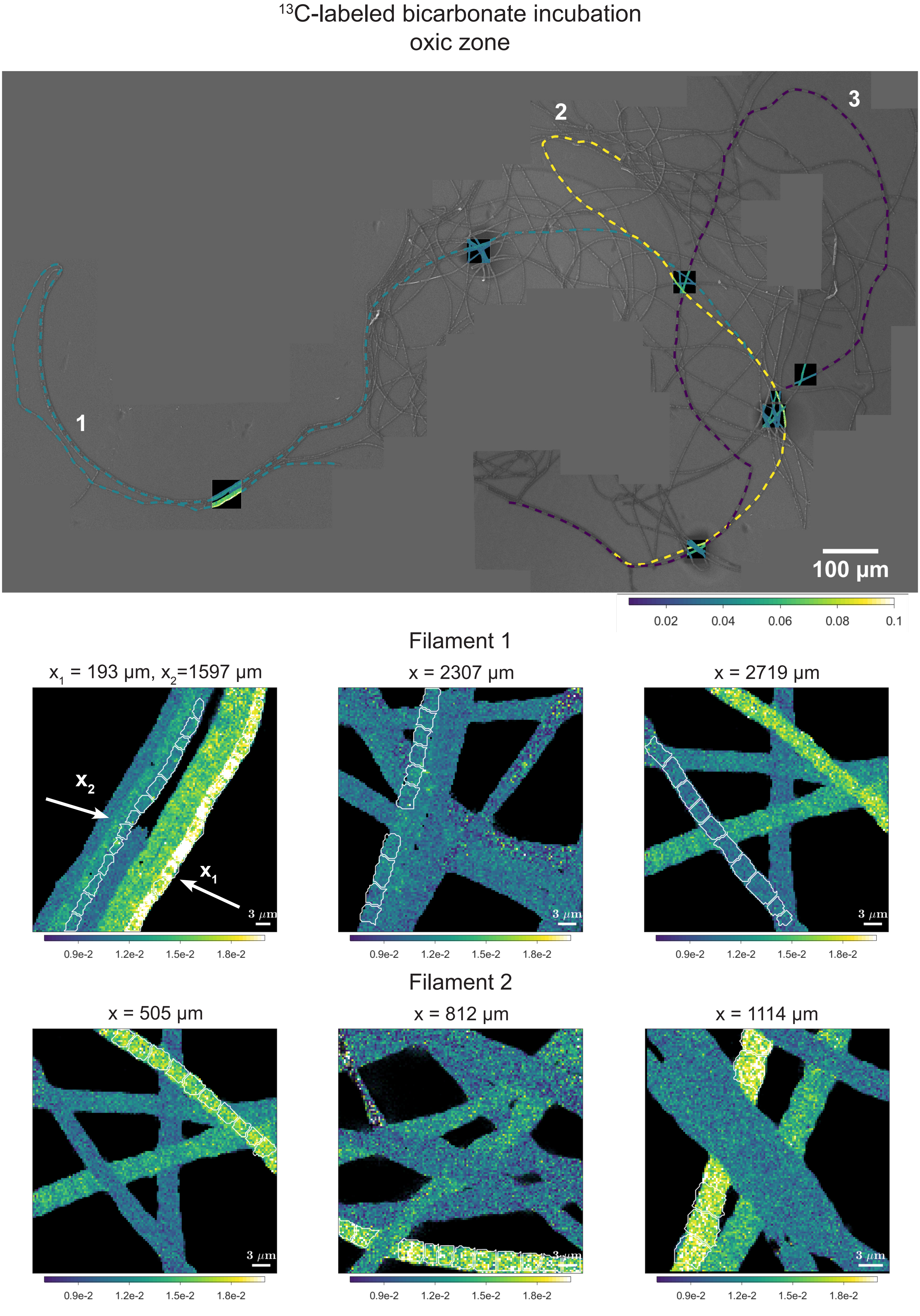

^{13}C-labeled bicarbonate incubation
oxic zone
1
2
3
100 µm
0.02
0.04
0.06
0.08
0.1
Filament 1
x_1 = 193 µm, x_2=1597 µm
x = 2307 µm
x = 2719 µm
x_2
x_1
3 µm
0.9e-2
1.2e-2
1.5e-2
1.8e-2
Filament 2
x = 505 µm
x = 812 µm
x = 1114 µm

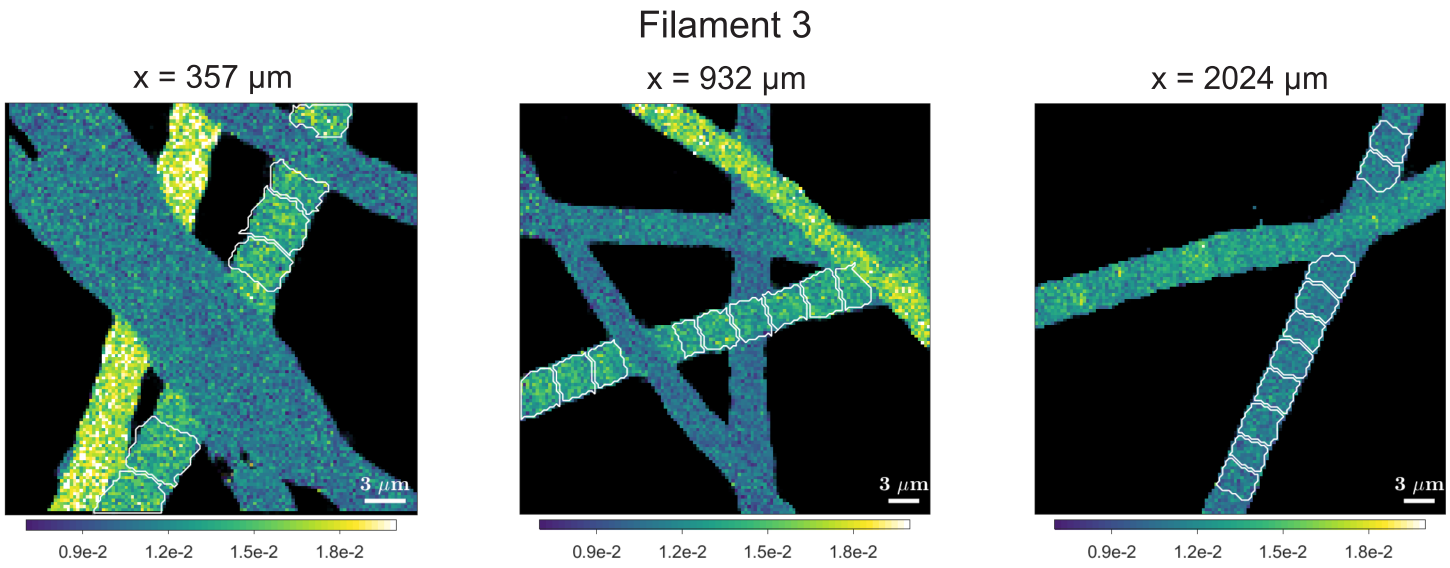
Filament 3
x = 357 μm
x = 932 μm
x = 2024 μm
3 μm
3 μm
3 μm
0.9e-2
1.2e-2
1.5e-2
1.8e-2
0.9e-2
1.2e-2
1.5e-2
1.8e-2
0.9e-2
1.2e-2
1.5e-2
1.8e-2

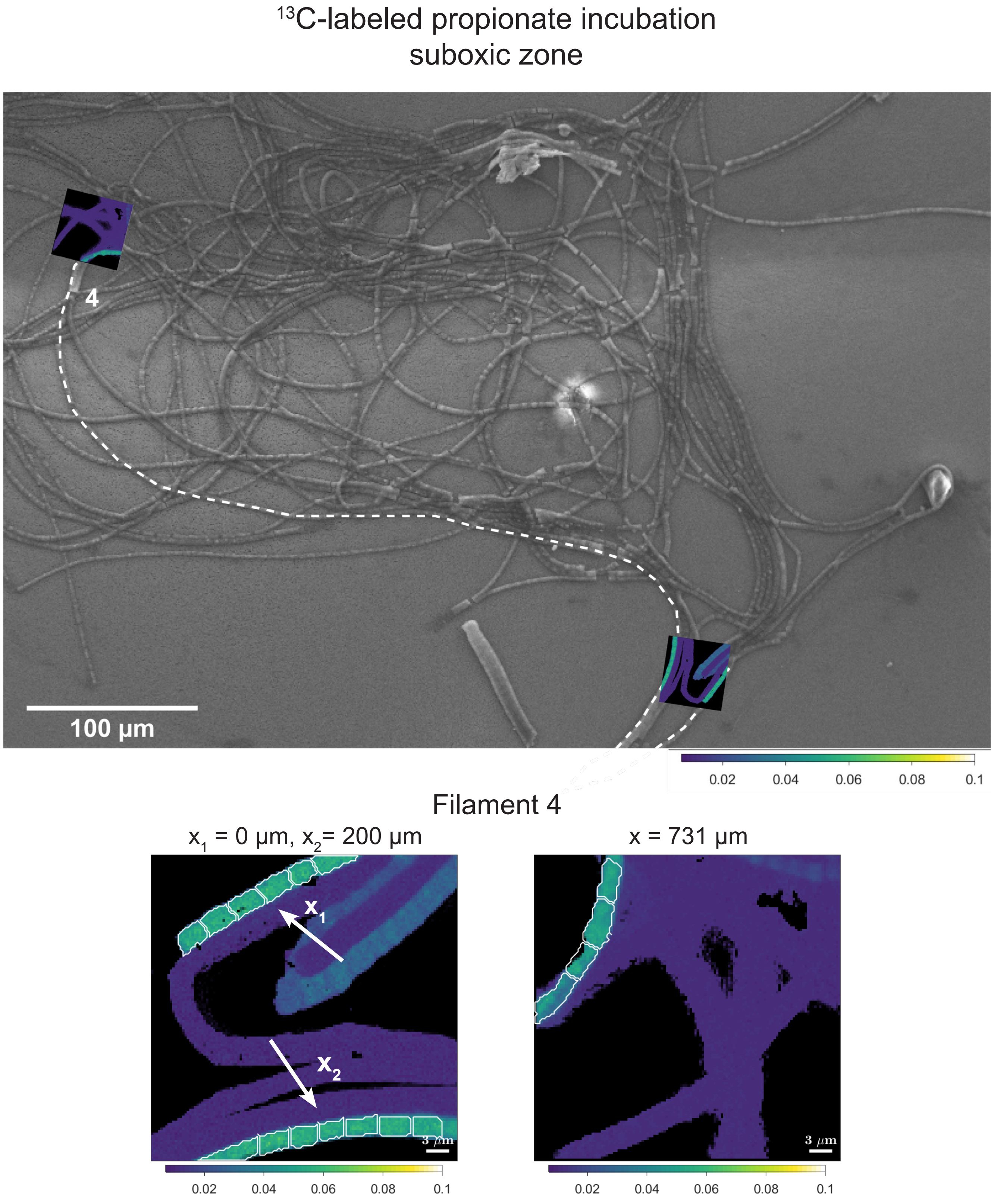

13C-labeled propionate incubation
suboxic zone
4
100 µm
0.02
0.04
0.06
0.08
0.1
Filament 4
x1 = 0 µm, x2= 200 µm
x = 731 µm
x1
x2
3 µm
3 µm

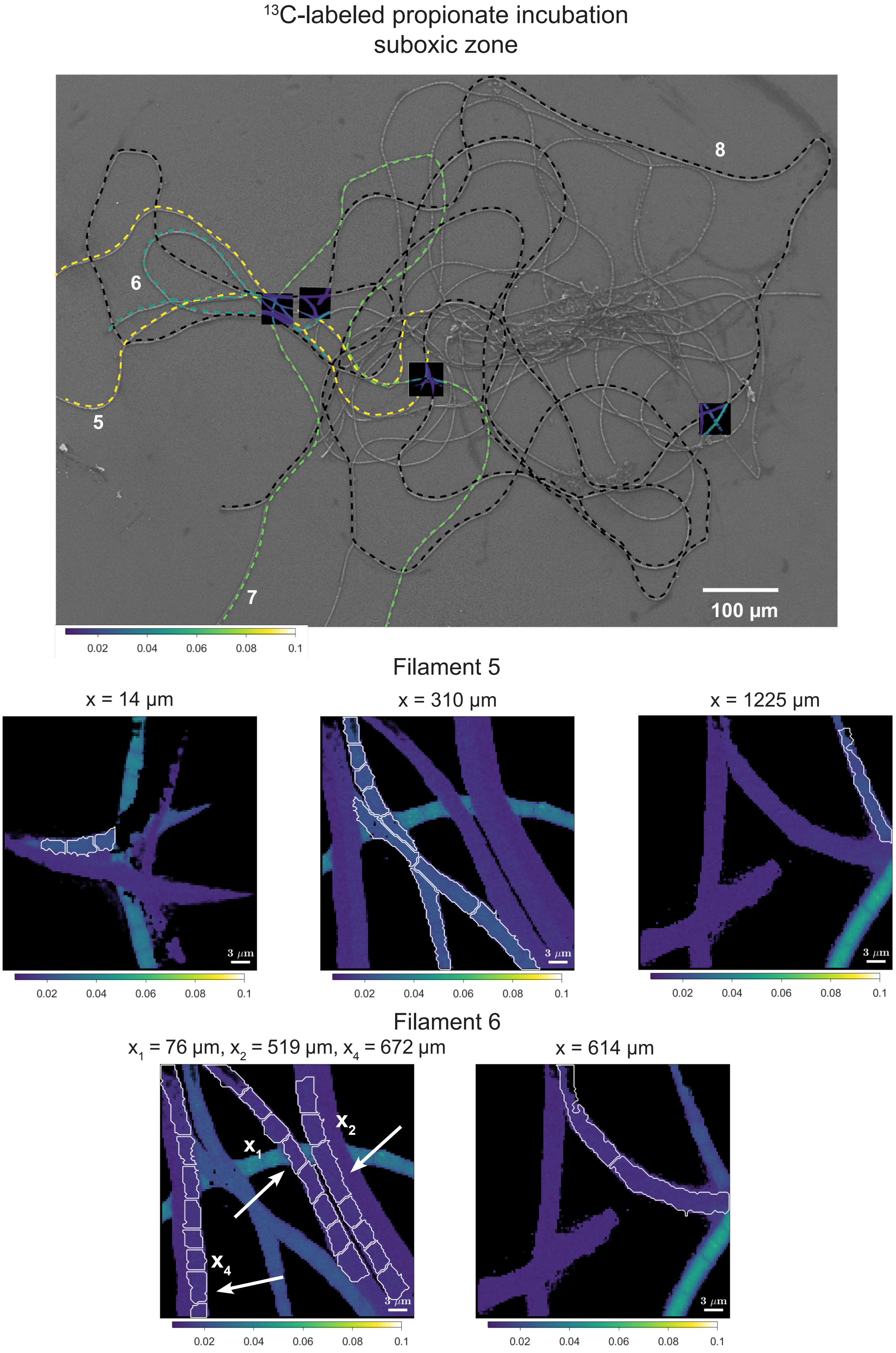

^{13}C-labeled propionate incubation
suboxic zone
8
6
5
7
100 µm
0.02 0.04 0.06 0.08 0.1
Filament 5
x = 14 µm
x = 310 µm
x = 1225 µm
3 µm
Filament 6
x_1 = 76 µm, x_2 = 519 µm, x_4 = 672 µm
x = 614 µm
x_1
x_2
x_4

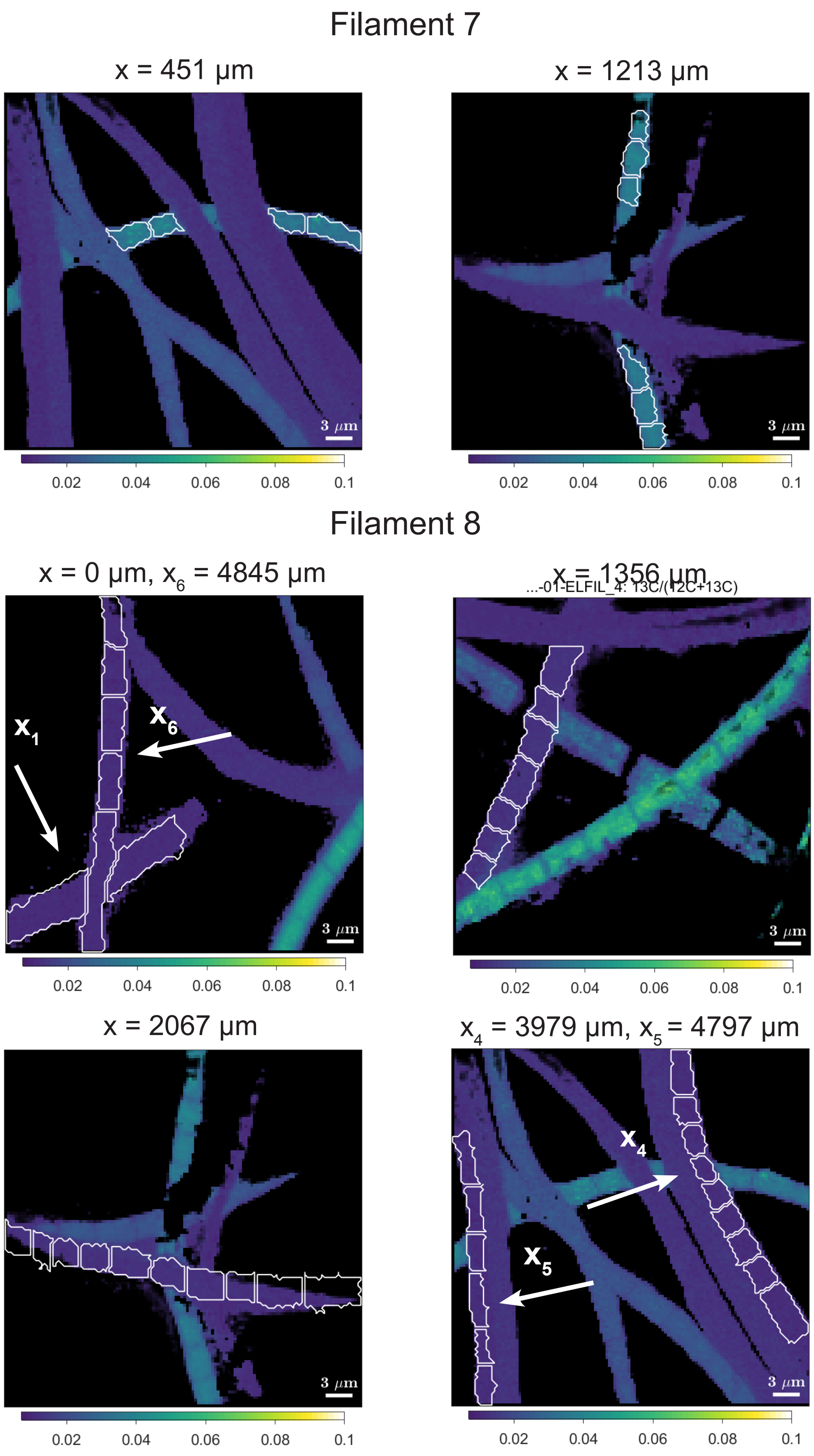

Filament 7
x = 451 μm
x = 1213 μm
3 μm
0.02 0.04 0.06 0.08 0.1
Filament 8
x = 0 μm, x6 = 4845 μm
x1
x6
x = 1356 μm
...-01-ELFIL_4: 13C/(12C+13C)
x = 2067 μm
x4 = 3979 μm, x5 = 4797 μm
x4
x5

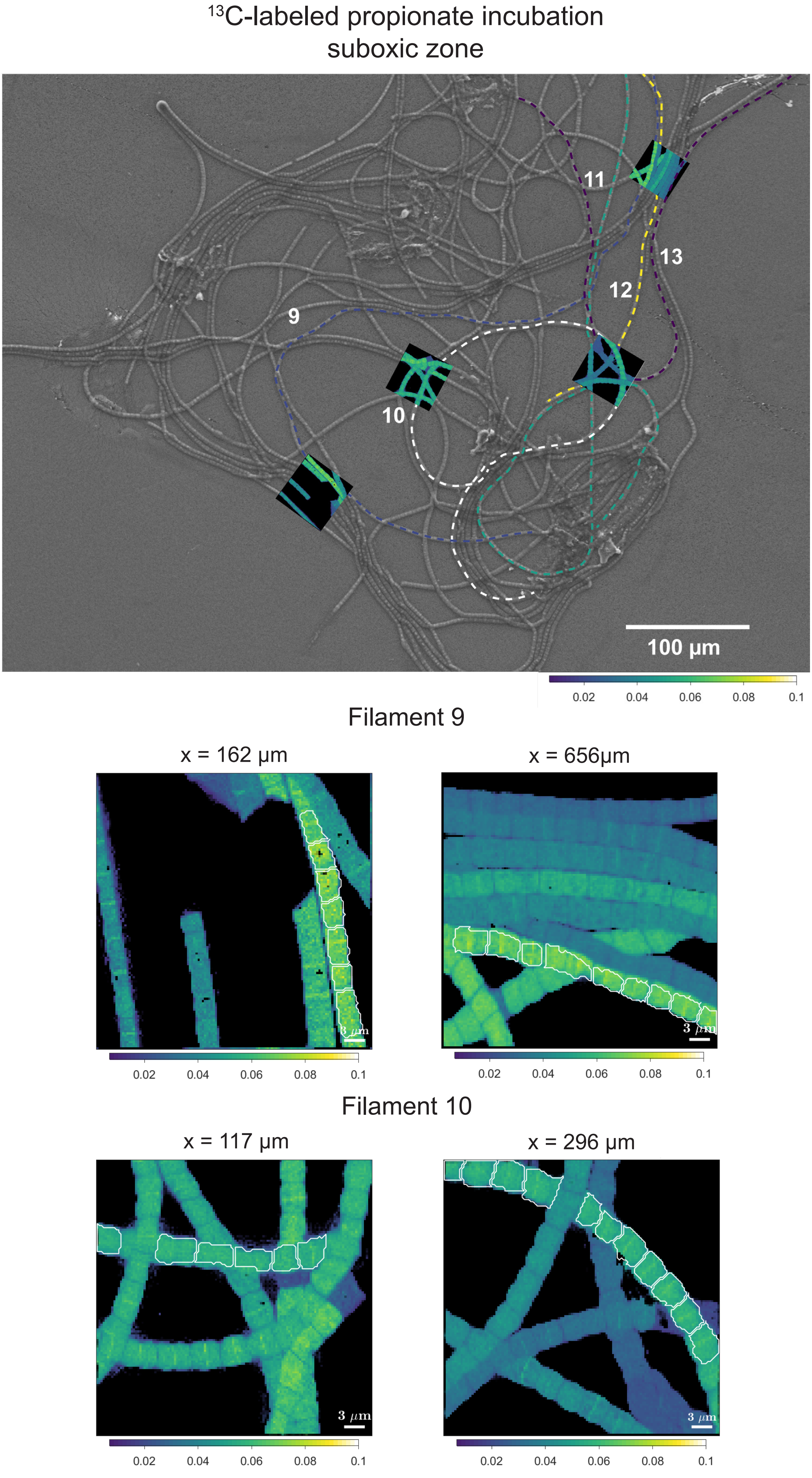

13C-labeled propionate incubation
suboxic zone
9
10
11
12
13
100 µm
0.02 0.04 0.06 0.08 0.1
Filament 9
x = 162 µm
x = 656µm
3 µm
Filament 10
x = 117 µm
x = 296 µm

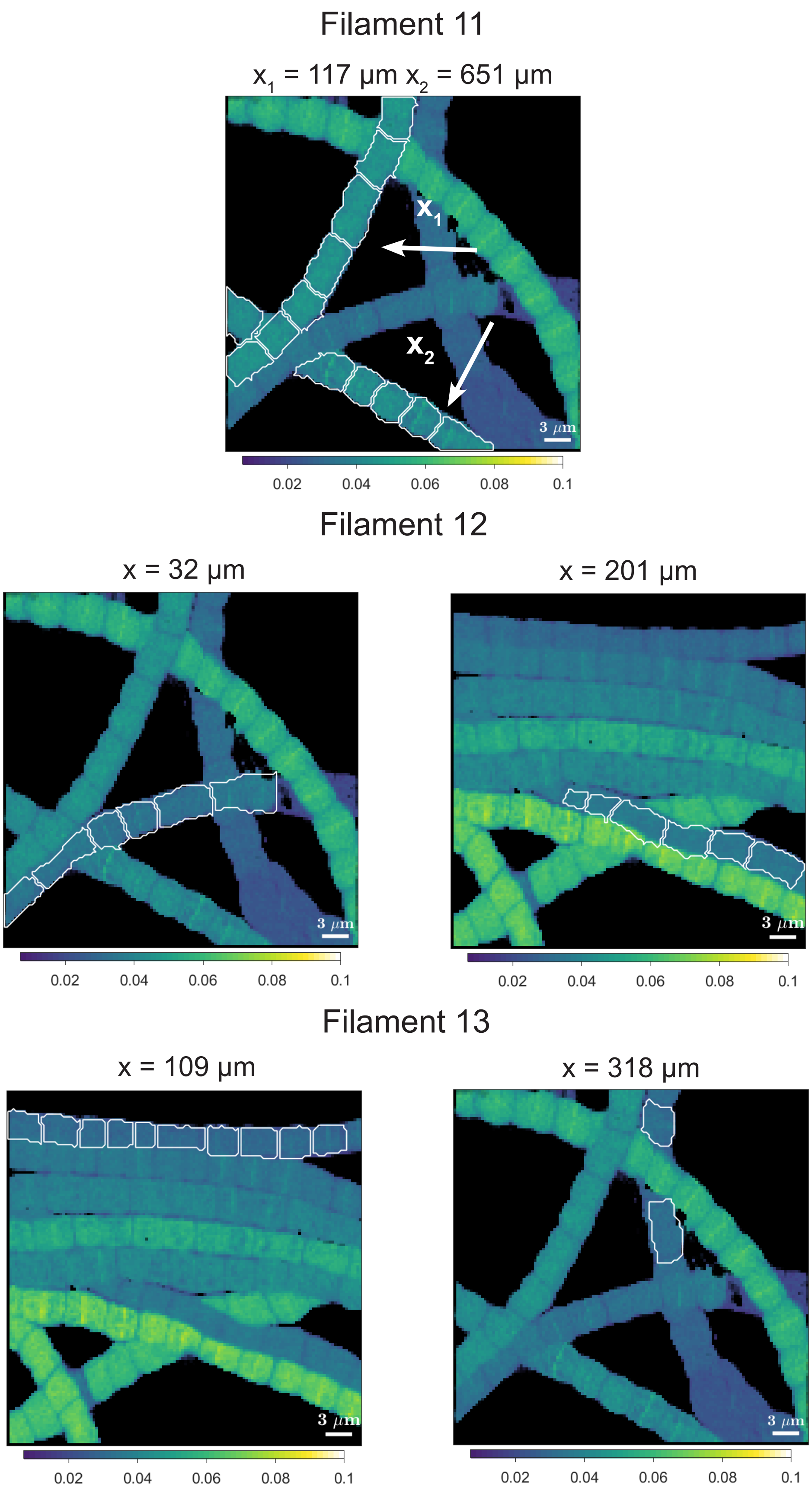
Filament 11
x_1 = 117 µm x_2 = 651 µm
x_1
x_2
3 µm
0.02 0.04 0.06 0.08 0.1
Filament 12
x = 32 µm
x = 201 µm
3 µm
3 µm
0.02 0.04 0.06 0.08 0.1
0.02 0.04 0.06 0.08 0.1
Filament 13
x = 109 µm
x = 318 µm
3 µm
3 µm
0.02 0.04 0.06 0.08 0.1
0.02 0.04 0.06 0.08 0.1

^{13}C-labeled bicarbonate incubation
suboxic zone

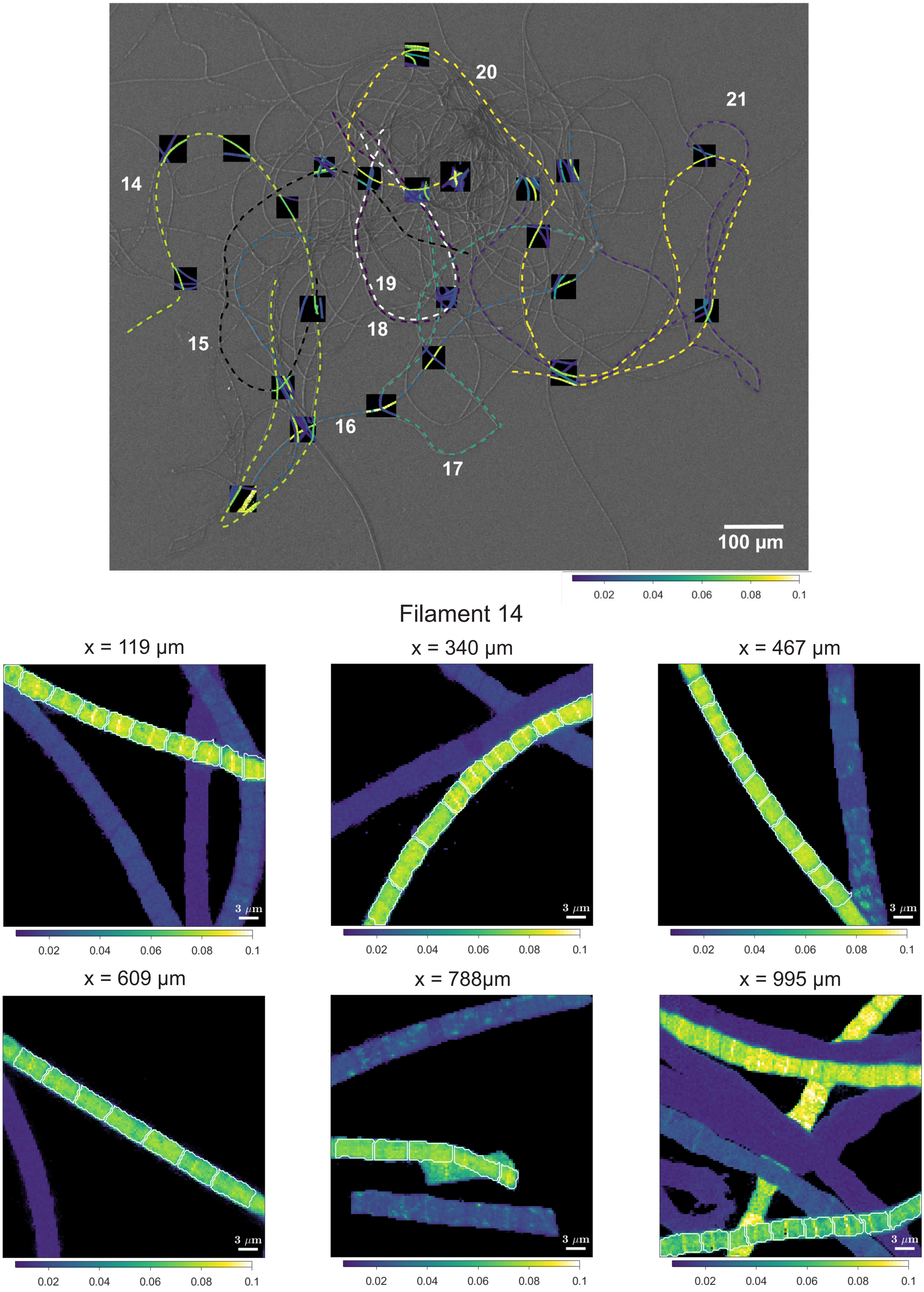

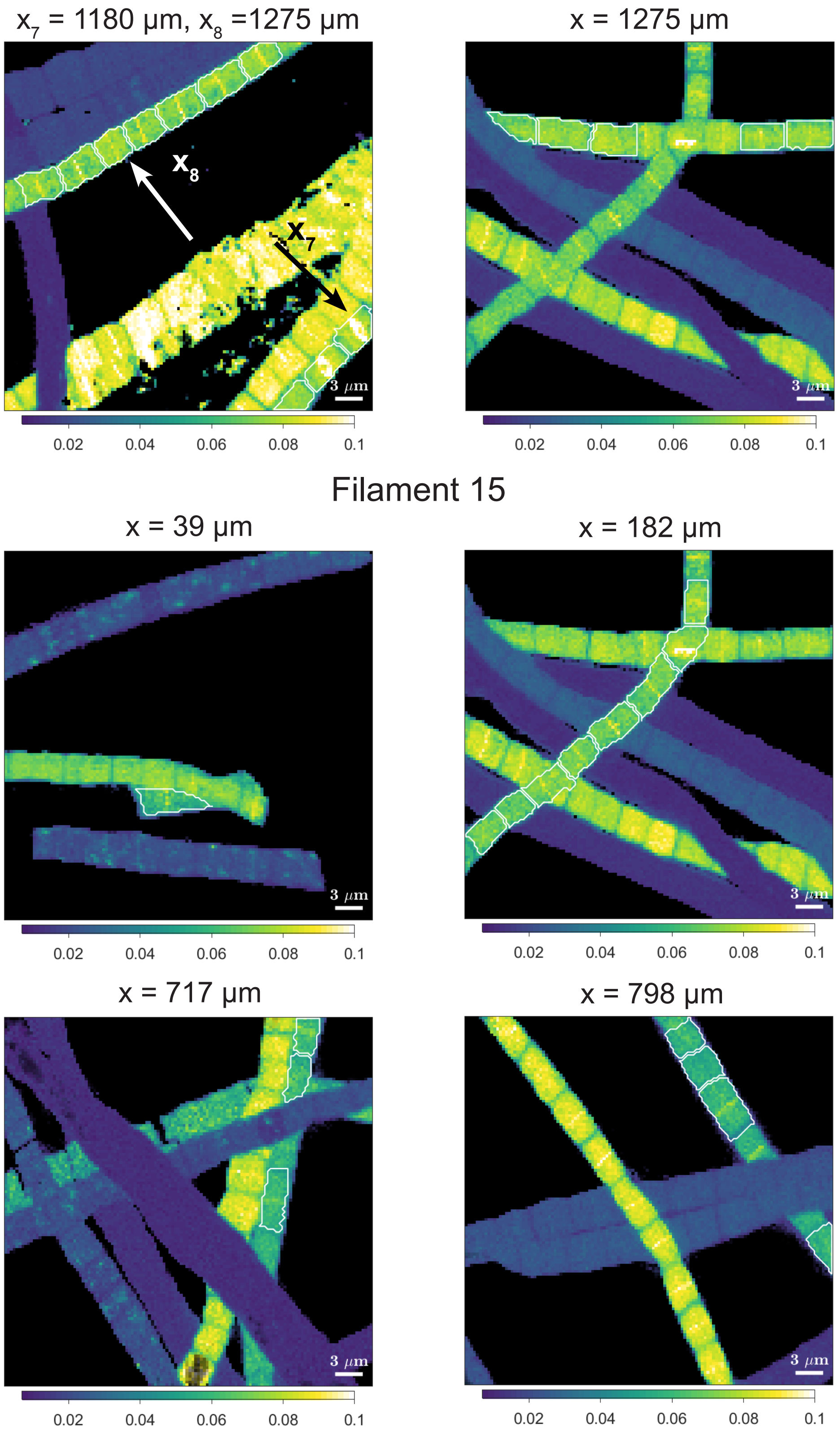

x_7 = 1180 μm, x_8 =1275 μm
x_8
x_7
3 μm
0.02 0.04 0.06 0.08 0.1
x = 1275 μm
3 μm
0.02 0.04 0.06 0.08 0.1
Filament 15
x = 39 μm
3 μm
0.02 0.04 0.06 0.08 0.1
x = 182 μm
3 μm
0.02 0.04 0.06 0.08 0.1
x = 717 μm
3 μm
0.02 0.04 0.06 0.08 0.1
x = 798 μm
3 μm
0.02 0.04 0.06 0.08 0.1

Filament 16

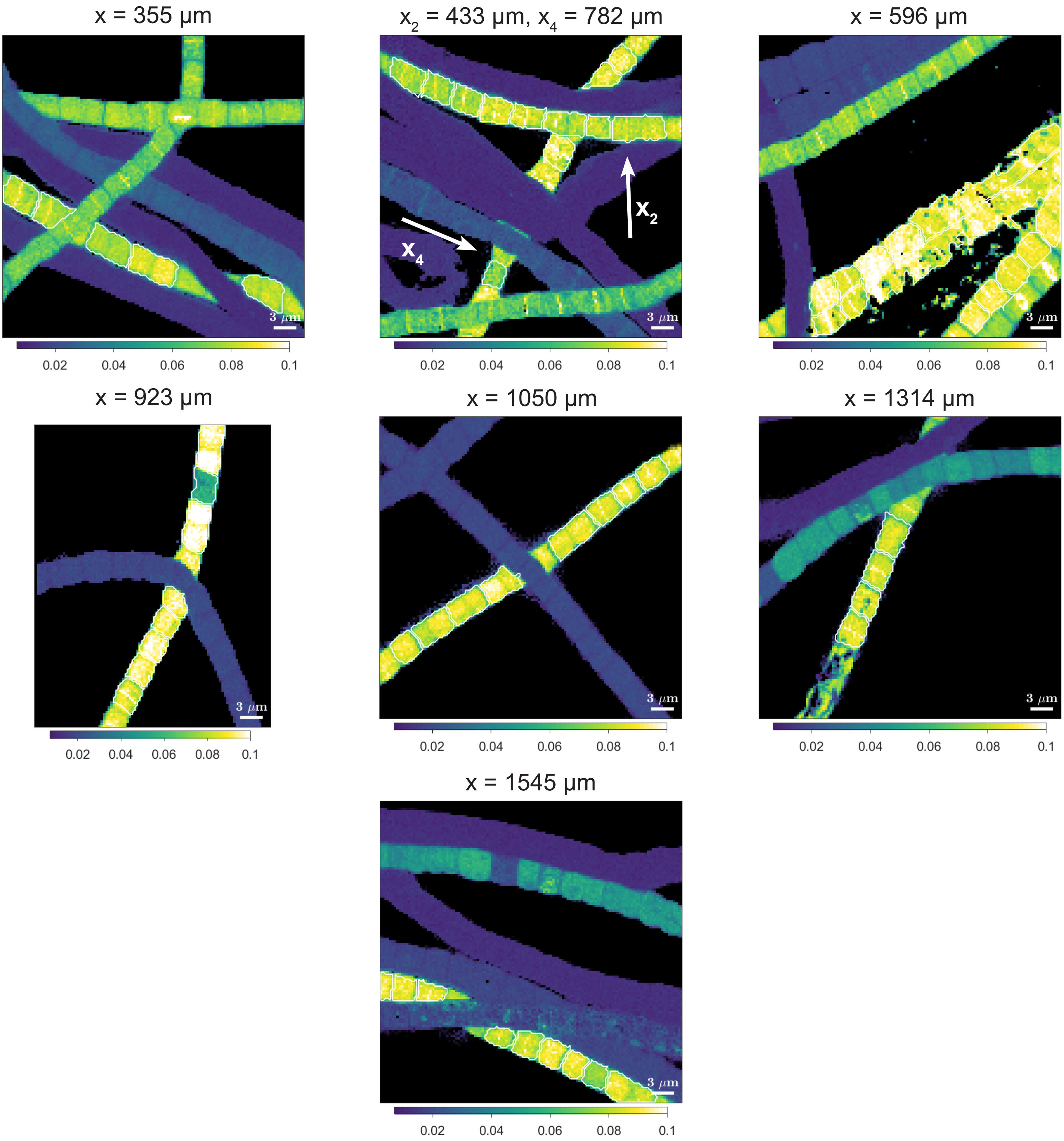

x = 355 μm
x₂ = 433 μm, x₄ = 782 μm
x = 596 μm
x = 923 μm
x = 1050 μm
x = 1314 μm
x = 1545 μm
x₂
x₄
3 μm
0.02
0.04
0.06
0.08
0.1

Filament 17

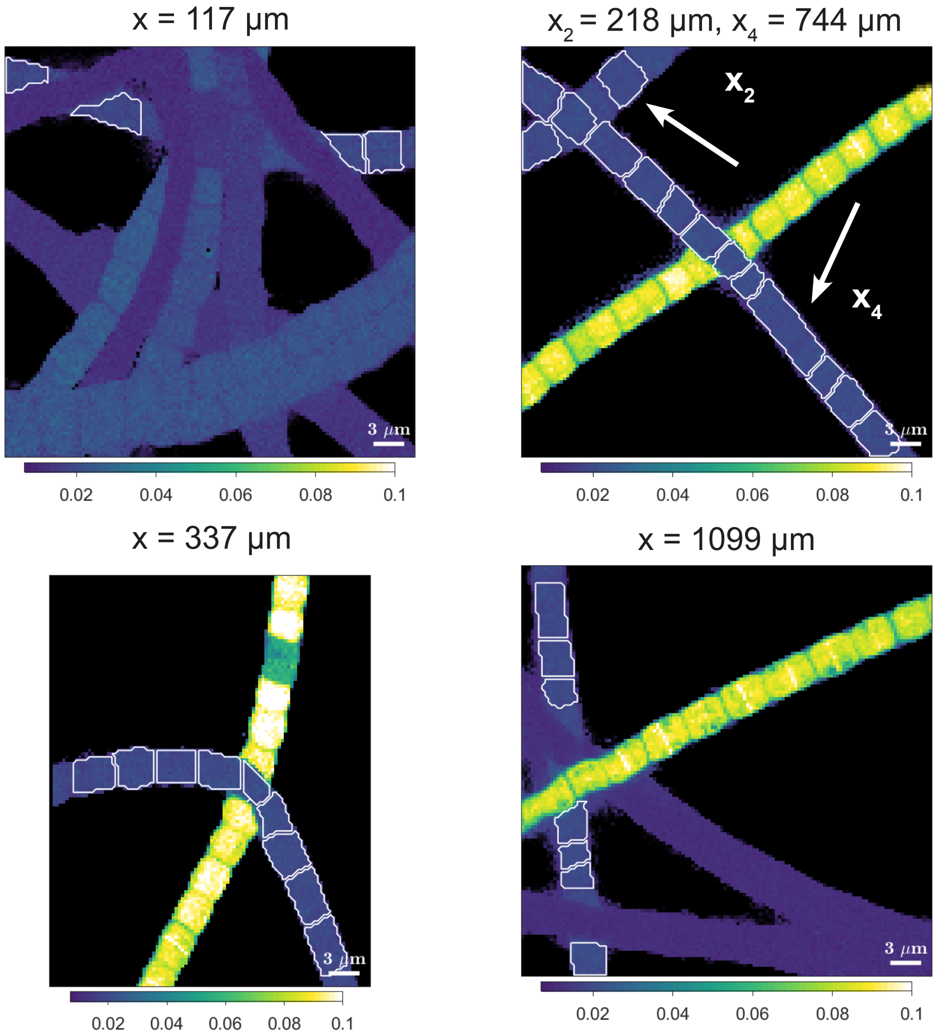

Filament 18

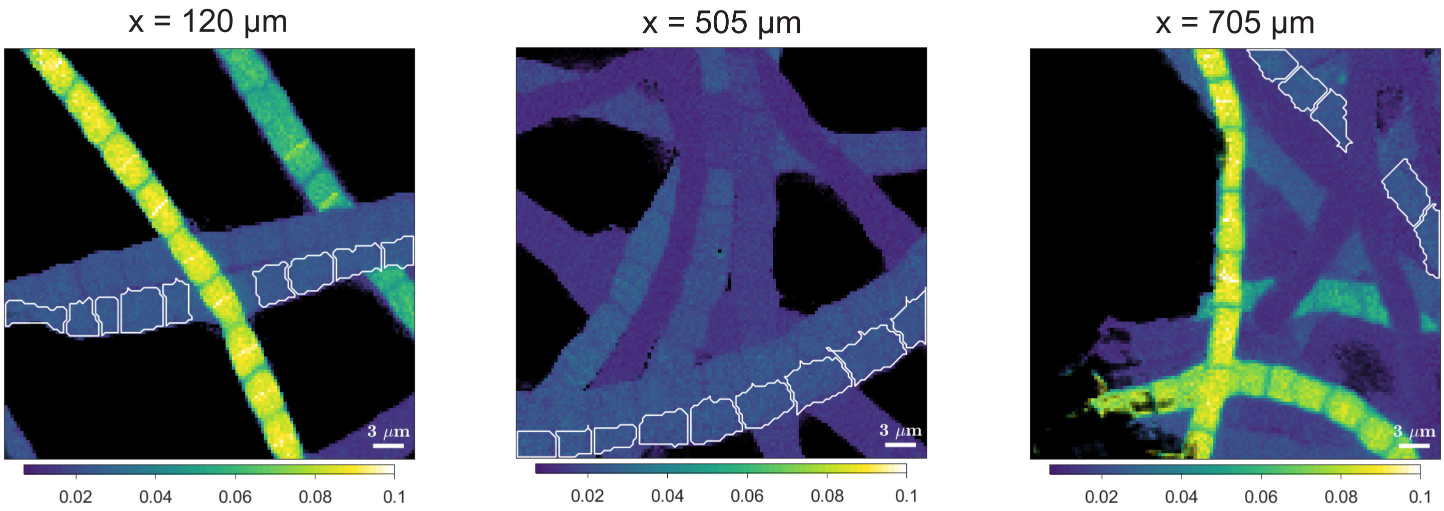

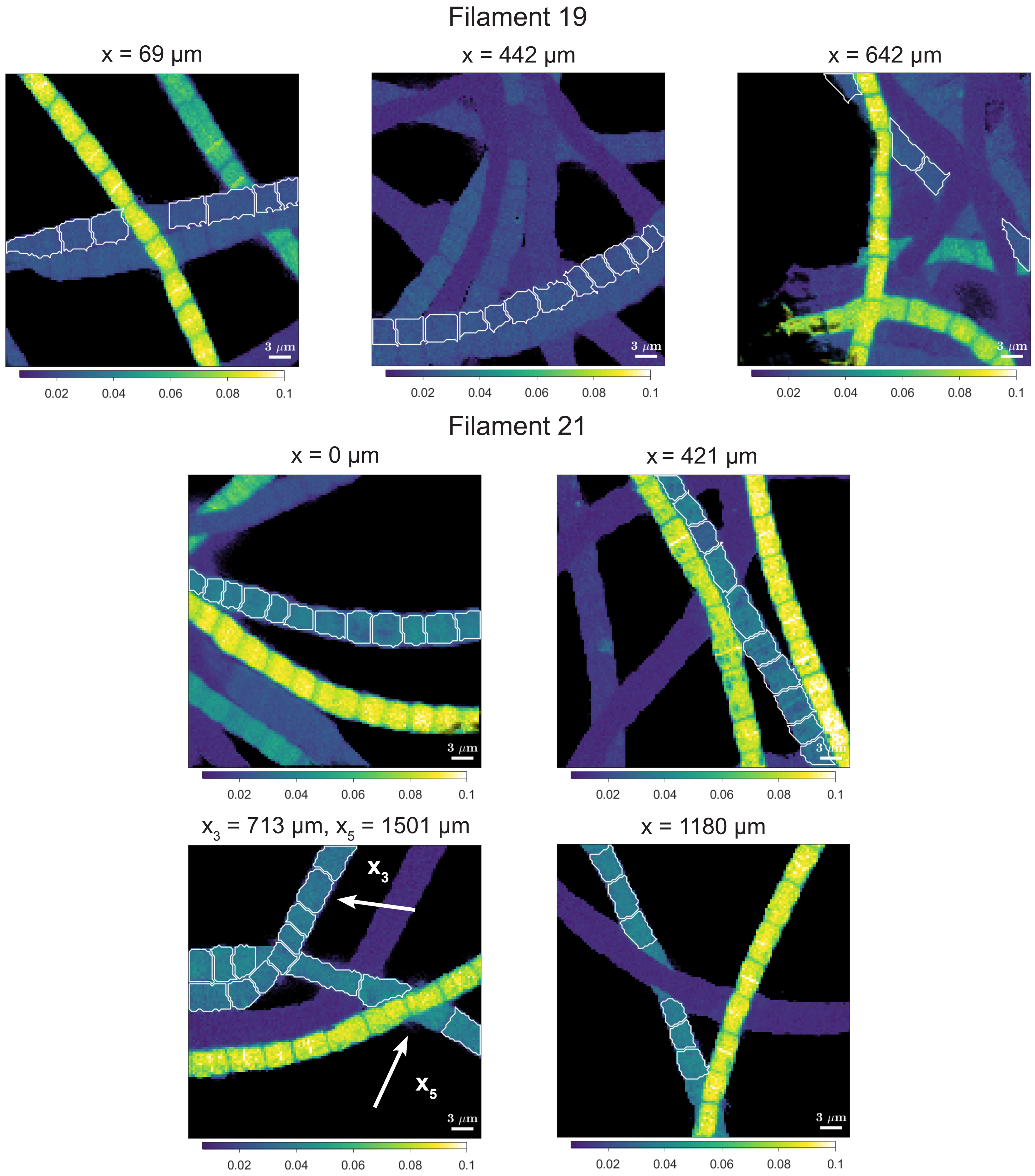
Filament 19
x = 69 μm
x = 442 μm
x = 642 μm
3 μm
0.02 0.04 0.06 0.08 0.1
Filament 21
x = 0 μm
x = 421 μm
x3 = 713 μm, x5 = 1501 μm
x = 1180 μm
x3
x5
