# Supplementary material for: Cell Cycle, Filament Growth and Synchronized Cell Division in Multicellular Cable Bacteria
Source: Front Microbiol. 2021 Jan 27;12:620807. doi: 10.3389/fmicb.2021.620807 (PMC7873302; doi:10.3389/fmicb.2021.620807)

Supplementary Methods

To gain insight into the 3D distribution of the isotopic composition within cells, the Look@NanoSIMS program (Polerecky et al., 2012) was updated by adding a new feature that allows visualization of the depth variation in the nanoSIMS data (ion counts or ion count ratios) along a lateral profile. A stepwise description of this feature is provided here. For clarity, screenshots are used. The LANS program can be downloaded from http://nanosims.geo.uu.nl/nanosims-wiki/doku.php/nanosims:lans

After starting LANS, the first step will load the raw data (file extension **im**) in blocks via the Look@NanoSIMS program. Select the file(s) that need to be analyzed. Once the files are selected a pop-up window will allow the selection of the block size.

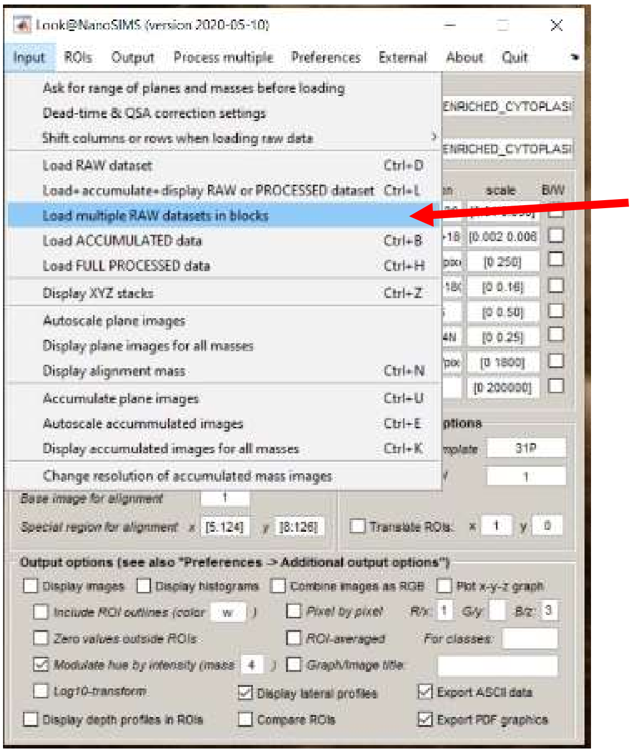

Once the block size is selected the program will load the raw data and automatically accumulate and align (i.e. drift-correction) the planes within a block. Once a block is accumulated and aligned it will be treated as a single plane. After a file is loaded in blocks, the program will ask anew how many planes are in a block for a next file. The block size can thus be changed per file which might be useful if conditions were changed between measurements (e.g. accumulation time). The MATLAB console will tell how far along the program is with the accumulation and alignment of the data.

After all planes are loaded in blocks and each block will be subsequentially treated as a plane, the separate blocks need to be aligned to one another and subsequently accumulated. This is done as decribed in Polerecky et al., (2012) using the *Accumulation options* box. The translations applied to the selected base mass will be applied to all other masses and stored in the **xyaling.mat** file (Polerecky et al., 2012).

After the selected planes are loaded in blocks, aligned an accumulated, it is possible to analyze the depth variation along selected lateral lines. Check *Display lateral profiles* in the *Output options* box. Depth analysis can be done both on masses and ion count ratios. Select *Display Masses* or *Display Rations* using the the *Output* menu.

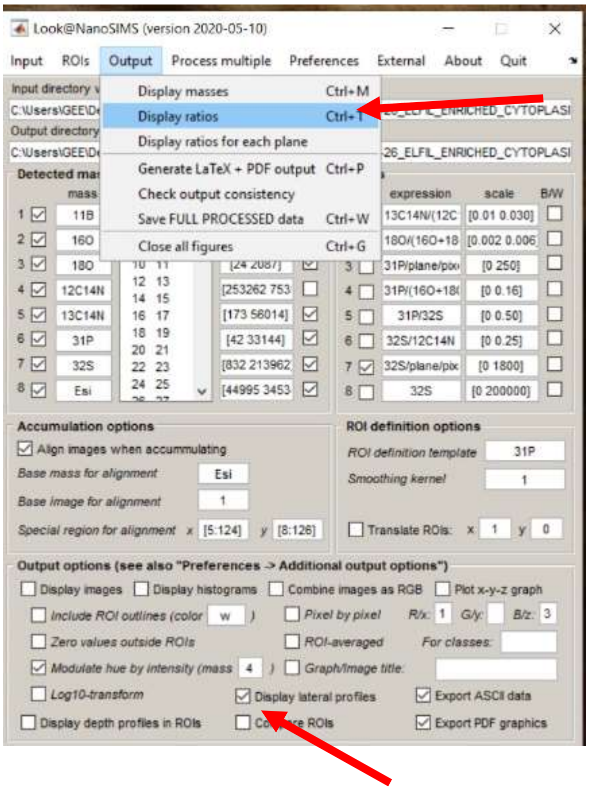

Selection of *Display Masses* or *Display Ratios* will open a new pop-up window. In this window the polyline is drawn along which the lateral depth profile will be analyzed. In this window the *Profile thickness* (in pixels) of the lateral profile and the *Image scale* can be adjusted. Use the *Draw polyline* button to draw the line along which the lateral profile need to be taken. This does not need to be a straight line. A single left-mouse click allows you to draw a point (Vertex). A line consisting of multiple points can be drawn. A double mouse click finishes the line.

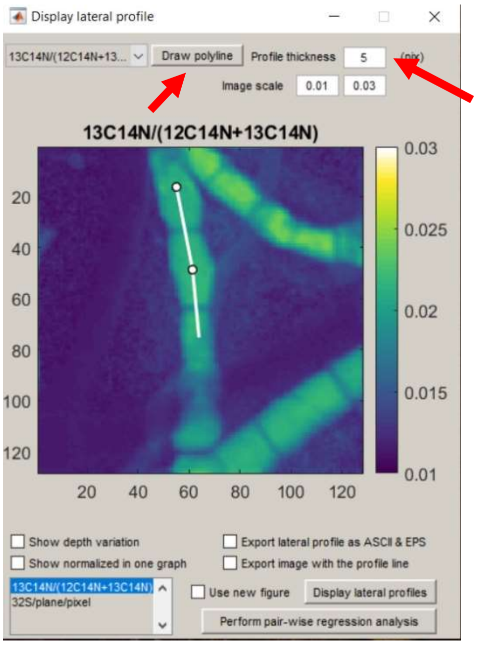

Once the polyline is drawn select in the menu below the image which figures need to be shown and which data needs to be exported. In the example below only the option *Show depth variation* was selected. The depth profile along the selected line is shown in another pop-up box.

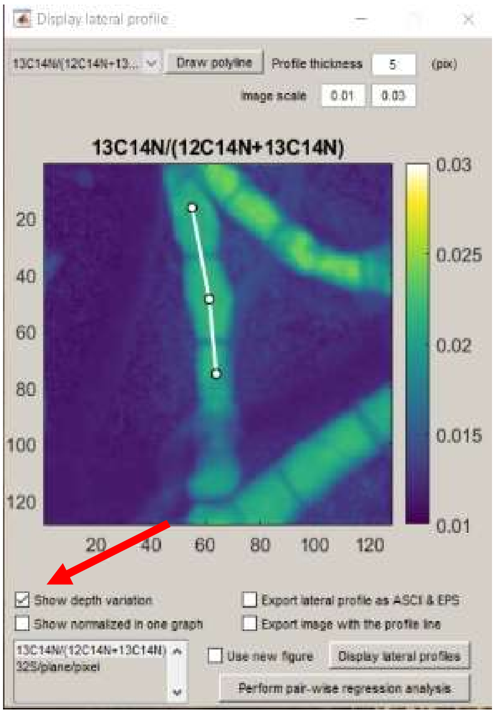

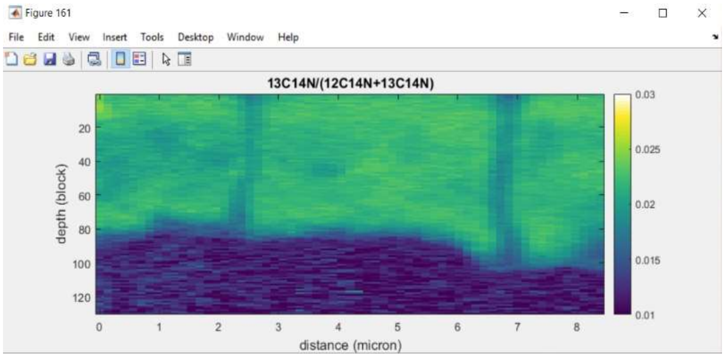

Supplement: Supplementary Method — Stepwise description of how to visualize the depth variation in the nanoSIMS data along a lateral profile using Look@NanoSIMS. [file Data_Sheet_1.PDF]
